# Availability, cost and affordability of essential medicines for chronic respiratory diseases in low-income and middle-income countries: a cross-sectional study

**DOI:** 10.1136/thorax-2023-221349

**Published:** 2024-05-17

**Authors:** Marie Stolbrink, Obianuju B Ozoh, David M G Halpin, Rebecca Nightingale, Jamilah Meghji, Catherine Plum, Brian William Allwood, Shamanthi Jayasooriya, Kevin Mortimer

**Affiliations:** 1 Clinical Sciences, Stellenbosch University, Stellenbosch, South Africa; 2 Clinical Sciences, Liverpool School of Tropical Medicine, Liverpool, UK; 3 Department of Medicine, University of Lagos College of Medicine, Lagos, Nigeria; 4 Department of Medicine, Lagos University Teaching Hospital, Surulere, Nigeria; 5 Department of Respirology, Royal Devon and Exeter Hospital, Exeter, UK; 6 Imperial College London, London, UK; 7 University Hospitals of Morecambe Bay NHS Trust, Kendal, UK; 8 Department of Pulmonology, Tygerberg Academic Hospital, Cape Town, South Africa; 9 Department of Pulmonology, Stellenbosch University Faculty of Medicine and Health Sciences, Cape Town, South Africa; 10 Academic Unit of Primary Care, The University of Sheffield, Sheffield, UK; 11 Department of Medicine, University of Cambridge, Cambridge, UK; 12 Department of Medicine, University of KwaZulu-Natal College of Health Sciences, Durban, South Africa; 13 Liverpool University Hospitals NHS Foundation Trust, Liverpool, UK

**Keywords:** Asthma, COPD Pharmacology, Asthma Pharmacology, Health Economist

## Abstract

Contemporary data on the availability, cost and affordability of essential medicines for chronic respiratory diseases (CRDs) across low-income and middle-income countries (LMICs) are missing, despite most people with CRDs living in LMICs. Cross-sectional data for seven CRD medicines in pharmacies, healthcare facilities and central medicine stores were collected from 60 LMICs in 2022–2023. Medicines for symptomatic relief were widely available and affordable, while preventative treatments varied widely in cost, were less available and largely unaffordable. There is an urgent need to address these issues if the Sustainable Development Goal 3 is to be achieved for people with asthma by 2030.

## Introduction

The forthcoming High-Level Meeting of the United Nations General Assembly on Non-Communicable Diseases (NCDs) will address the 15 million premature deaths from NCDs annually, most of which occur in low-income and middle-income countries (LMICs).^1^ The United Nations Sustainable Development Goals (SDGs) demand ‘safe, effective, quality and affordable essential medicines for all’ by 2030.^2^ Non-communicable chronic respiratory diseases (CRDs), for example, asthma and chronic obstructive pulmonary disease (COPD), cause substantial morbidity and mortality, disproportionately affecting those living in poverty in LMICs.^3^ Recommended medicines for CRDs include inhaled and oral drugs that are on the WHO Model List of Essential Medicines (EML), which defines safe, efficacious, cost-effective medicines that should be available everywhere.^4^ Access to essential, affordable CRD medicines is limited in LMICs.^5 6^ Up-to-date data on the availability, cost and affordability of WHO essential medicines for CRDs in LMICs are missing.

## Methods

This cross-sectional survey of medication availability and cost was completed by healthcare professionals working in LMICs (online supplemental appendix). Professionals collected standardised data from three facilities (pharmacy, healthcare facility (HCF) and central medicine store (CMS)) in each country. Prices (US$) for 1-month treatment were compared. Affordability was defined by 1-month treatment costing the lowest paid government worker <1 day’s wage, as per established methodology.^7^

10.1136/thorax-2023-221349.supp1 Supplementary data

## Results

Data from 60 LMICs were collected between June 2022 and April 2023 (online supplemental appendix). 18 countries were low income, 24 lower middle income and 17 upper middle income. The sub-Saharan African region was best represented (27 countries). Information for all three facilities was submitted for 42/60 (70%) countries. Information for pharmacies, HCFs and CMS was provided by 57, 56 and 46 LMICs, respectively. Most pharmacies were private (89%), most HCFs were public institutions (80%).

### Inhaled short-acting beta agonists

**Figure 1:**
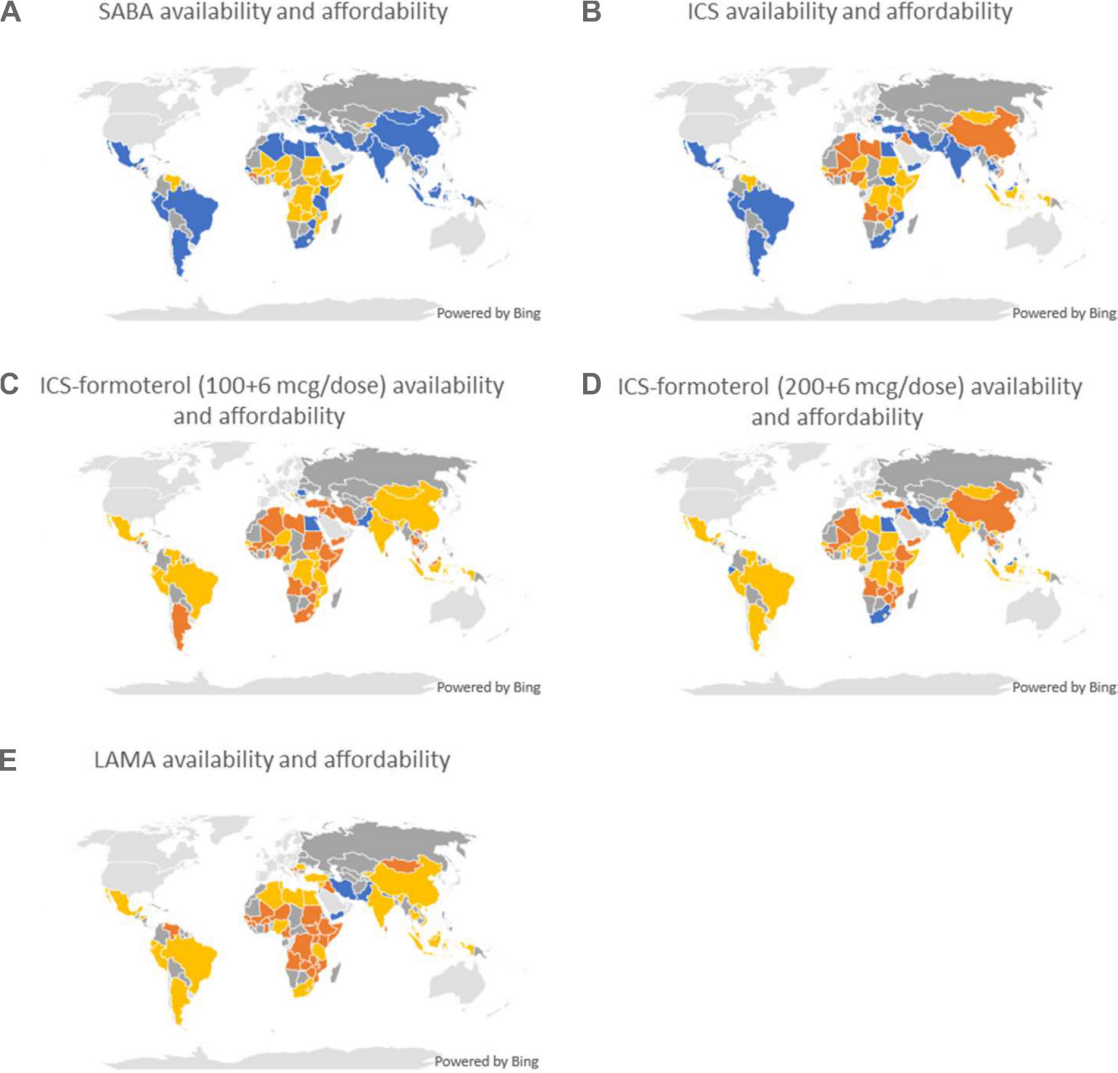
Availability and affordability of standardised (A) SABA, (B) ICS, (C) ICS-LABA (100+6 mcg/dose), (D) ICS-LABA (200+6 mcg/dose) and (E) LAMA formulations in pharmacy or HCF. Blue: available and affordable. Yellow: available but not affordable. Orange: unavailable. Grey: low-income and middle-income country (LMIC), no information. HCF, healthcare facility; ICS, inhaled corticosteroid; ICS-LABA, inhaled corticosteroid–long-acting beta agonist (formoterol) combination; LAMA, long-acting muscarinic antagonist inhaler; SABA, short-acting beta agonist inhaler.

Inhaled short-acting beta agonist (SABA) was available in 93% of pharmacies, 79% of HCFs and 78% of CMS (table 1, figure 1, online supplemental appendix). The median cost of 1-month treatment in pharmacies was $2.95 (IQR $1.99–4.97), $2.34 (IQR $1.38–3.86) in HCFs and $1.39 (IQR $1.20–2.83) in CMS. SABAs were both available and affordable in 51% (29/57) of all pharmacies and 61% (31/56) of all HCFs that submitted data.

**Table 1:** Comparisons of availability and costs for 1-month treatment for standardised SABA, ICS, ICS-LABA and LAMA formulations in pharmacy, HCF and CMS

Facility	Medicine					
	SABA	ICS	ICS-LABA (100+6 mcg/dose)	ICS-LABA (200+6 mcg/dose)	LAMA	5-day oral OCS*
Pharmacy						
Availability	53/57 (93%)	31/57 (54%)	22/57 (39%)	31/57 (54%)	26/57 (46%)	47/57 (82%)
Median cost (IQR, US$)	2.95 (1.99–4.97)	5.40 (2.12–8.60)	19.71 (12.00–42.00)	19.20 (9.73–27.43)	30.53 (9.45–47.29)	1.65 (0.60–3.29)
Median DOW (IQR)	0.8 (0.2–1.8)	0.6 (0.3–3.5)	4.3 (2.6–8.3)	4.1 (1.9–6.9)	3.5 (1.4–5.8)	0.3 (0.1–1.0)
HCF						
Availability	44/56 (79%)	31/56 (55%)	10/56 (18%)	21/56 (38%)	16/56 (29%)	40/56 (71%)
Median cost (IQR, US$)	2.34 (0.1–1.0)	3.01 (1.21–5.89)	16.49 (12.03–26.55)	18.41 (11.30–24.49)	26.01 (15.32–36.70)	0.02 (0.01–0.08)
Median DOW (IQR)	0.4 (0.1–1.9)	0.5 (0.1–1.8)	3.2 (2.2–4.4)	3.5 (1.5–6.1)	3.3 (1.4–5.6)	0.01 (0–0.03)
CMS						
Availability	36/46 (78%)	22/46 (48%)	7/46 (15%)	9/46 (20%)	11/46 (24%)	35/46 (76%)
Median cost (IQR, US$)	1.39 (1.20–2.83)	1.16 (0.11–3.24)	26.48 (13.31–29.85)	7.14 (3.90–8.13)	17.98 (0.98–32.17)	0.02 (0.01–0.03)

Availability: number of facilities where medicine is available by total number of facilities that submitted data. See the online supplemental appendix for definitions of standardised formulations.

*Standardised formulation for OCS is 5-day course of oral prednisolone, 40 mg once a day using 5 mg tablets. CMS costs are wholesale costs, unsuitable for affordability/days of work calculations.

CMS, central medicine stores; DOW, days of work required to pay for 1-month treatment; HCF, healthcare facility; ICS, inhaled corticosteroid; ICS-LABA, inhaled corticosteroid–long-acting beta agonist (formoterol) combination; LAMA, long-acting muscarinic antagonist inhaler; OCS, oral corticosteroids; SABA, short-acting beta agonist inhaler.

### Inhaled corticosteroids

Beclomethasone 100 mcg/dose, or equivalent, was available in 54% of pharmacies, 55% of HCFs and 48% of CMS (table 1, figure 1, online supplemental appendix). The median costs were $5.40 (IQR $2.12–8.60) in pharmacies, $3.01 (IQR $1.21–5.89) in HCFs and $1.16 (IQR $0.11–3.24) in CMS. Inhaled corticosteroids (ICS) were both available and affordable in 30% (17/57) of pharmacies and 36% (20/56) of HCFs.

### Combination inhaled corticosteroid–long-acting beta agonists

Budesonide-formoterol 200+6 mcg/dose, or equivalent, was available in 54% of pharmacies, 38% of HCFs and 20% of CMS (table 1, figure 1, online supplemental appendix). The median costs were $19.20 (IQR $9.73–27.43) in pharmacies, $18.41 (IQR $11.30–24.49) in HCFs and $7.14 (IQR $3.90–8.13) in CMS. Overall, it was both available and affordable in 11% (6/57) of pharmacies and 5% (3/56) of HCFs. Costs for budesonide-formoterol 100+6 mcg/dose were similar, but it was less available.

### Long-acting anti-muscarinic antagonists

A standardised long-acting muscarinic antagonist (LAMA) formulation was available in 46% of pharmacies, 29% of HCFs and 24% of CMS (table 1, figure 1, online supplemental appendix). The median costs were $30.53 (IQR $9.45–47.29) in pharmacies, $26.01 (IQR $15.32–36.70) in HCFs and $17.98 (IQR $0.98–32.17) in CMS. LAMAs were available and affordable in 7% (4/57) of pharmacies and 4% (2/56) of HCFs.

### Other essential medicines, WHO regions and income groups

Oral prednisolone was affordable and available between 70% and 80% of facilities (table 1). Other medicines were less available (online supplemental appendix). There were variations which region and income groups had the cheapest and most affordable medicines across facilities.

## Discussion

This is the largest cross-sectional study of availability, cost and affordability of CRD medicines in LMICs to date. It included data from 60 LMICs, representing 84% of the global LMIC population, and 16 of the 20 most populous LMICs.

Inhaled SABA and prednisolone were almost universally available. There were large cost ranges, SABAs were the cheapest inhalers. Medicines were cheaper in HCFs than pharmacies and price typically increased between CMS and pharmacy/HCF. In some countries medicines were free or subsidised.

We identified improvement in inhaled corticosteroid–long-acting beta agonist (ICS-LABA) availability compared with the last decade, with ICS-LABA now being available in more than half of pharmacies, and one-third of HCFs, possibly because of inclusion in the WHO EML and international guidelines.^6^ SABA and ICS availability in HCFs and CMS also improved compared with previously.^5^

Acute, symptomatic treatments (SABA, prednisolone) for CRDs were more affordable than daily treatments needed to reduce morbidity and mortality (ICS, ICS-LABA, LAMA). Guidelines recommend that first-line asthma treatment should be ICS-LABA, or ICS whenever SABA is taken.^8^ However, our findings suggest that these recommendations are largely unaffordable, and economic realities may force patients to use cheaper, riskier approaches.^8^

The median daily wage was $4.33 (IQR $2.17–9.55) providing an indication of ‘affordability’ for a month’s treatment, but acknowledging that many earn less than the lowest paid government worker. Making ICS-formoterol (median cost at least $16.49) affordable like this, or by benchmarking against SABA, could achieve a tipping point for the widespread adoption of anti-inflammatory reliever asthma therapy, especially considering the greater efficacy of ICS-formoterol.^8^ A similar approach to LAMA pricing could improve COPD management.^9^

Our study had several strengths. Data came from all income levels and regions and represented a large proportion of those living in LMICs. Comparisons were possible by using standardised data collection derived from established tools. Patient experience was reflected, as we presented a snapshot of the facility on the data collection day.

The study had some weaknesses. It was conducted over 11 months. Costs were compared using US$, dependent on exchange rates. Median price ratios were previously used for benchmarking, but the reference prices are out of date and no longer recommended.^7^ Convenience sampling introduced possible selection bias towards better equipped facilities in urban areas. Mostly only one facility was sampled per country. We did not assess medicine quality, expiry date nor data from multiple time points.

Establishing national CRD strategies that include medicines, generating country-specific data, buy-in from global organisations and patient advocacy are key to improving medicine access by addressing in-country demand and political commitment.^10^ Cost-effectiveness data for inhaled medicines specific for LMICs are needed, given that they reduce exacerbations and hospitalisations, which substantially drive the costs of CRDs.^9^ There is an urgent need to address the availability and affordability of essential CRD medicines if SDGs are to be achieved for all by 2030.
