# Supplementary material for: Availability, cost and affordability of essential medicines for chronic respiratory diseases in low-income and middle-income countries: a cross-sectional study
Source: Thorax. 2024 May 17;79(7):676–9. doi: 10.1136/thorax-2023-221349 (PMC11187362; doi:10.1136/thorax-2023-221349)
Supplement: Supplementary data [file thorax-2023-221349supp001.pdf]

Appendix – Availability, cost, and affordability of essential medicines for chronic respiratory diseases in low- and middle-income countries: a cross-sectional study – Stolbrink et al.

Appendix

Table of Contents

Table of Contents 1
Full Methods 2
Supplementary Table 1: Chronic Respiratory Diseases (CRDs) Medicines Survey Investigators 4
Supplementary Table 2: STROBE Statement[1] 7
Supplementary Table 3: Overview of included LMICs and facilities by income group and WHO region 9
Supplementary Table 4: Availability, cost, and affordability of SABA by country and type of facility. 11
Supplementary Table 5: Availability, cost, and affordability of ICS by country and type of facility. 14
Supplementary Table 6: Availability, cost, and affordability of ICS-LABA (200+6mcg/dose) by country and type of facility 17
Supplementary Table 7: Availability, cost, and affordability of ICS-LABA (100+6mcg/dose) by country and type of facility 20
Supplementary Table 8: Availability, cost, and affordability of LAMA by country and type of facility. 23
Supplementary Table 9: Availability and cost for one month's treatment for other essential medicines 26
Supplementary Figure 1: World map of LMICs where medicine data was available 27
Supplementary Figure 2: Box and whisker plots for ranges, interquartile ranges and medians of median 1) costs for one month's treatment in US$ in pharmacies and HCFs (a, b) and 2) days of work (DOW) for one month's treatment in pharmacies and HCFs (c, d). 28
Supplementary Figure 3: Median costs for one month's treatment in US$ or days of work for one month's treatment by WHO region and World Bank Income Group, in pharmacies (a-d) and HCF (e-h). 30
Supplementary Figure 4: Comparison of originator and generic medicine costs 32
References 33

Stolbrink M, *et al. Thorax* 2024; 79:676–679. doi: 10.1136/thorax-2023-221349

Appendix – Availability, cost, and affordability of essential medicines for chronic respiratory diseases in low- and middle-income countries: a cross-sectional study – Stolbrink et al.

Full Methods

Study design and data collection

This was a cross-sectional survey of medication availability and cost which was completed by healthcare professionals working in LMICs in 2022-2023. We followed STROBE guidelines and gained ethical approval from Liverpool School of Tropical Medicine (Supplementary data).[1] Investigators completed a standardised electronic data collection form covering demographics, availability and costs of essential medicines using kobotoolbox.[2] All investigators gave informed consent and gained local approval where necessary.

The data collection form used was based on established WHO methodology.[3,4] Essential medicines were defined by the WHO EML (Table 1).[5] Availability, strength, pack size and cost were recorded for generic and originator essential medicines. Originator brands were defined by the WHO "Database of medicine prices, availability, affordability and price components" as the products that were first authorised for marketing worldwide.[4] Investigators could also manually add medicines. Investigators entered the price that a patient would pay in the pharmacy or healthcare facility (e.g. clinic, hospital; HCF) that day for the cheapest product. Prices for the central medicine store or national procurement centre (CMS) were usually wholesale prices, and hence affordability was not calculated, as patients would be unable to purchase at this price themselves. Investigators provided photos to verify medicines and prices.

Table 1: Standardised doses and preparations for one month's treatment for essential medicines for chronic respiratory diseases, derived from WHO EMLO[5]

*Originator: product that was first authorised for marketing worldwide; (1): Inhaled beclomethasone and budesonide were considered equipotent.

Product	Drug class	Preparation	Standardised preparation for one month's treatment
Salbutamol	Short-acting beta-agonist (SABA)	100 mcg / dose, inhaler<br>5 mg / ml, nebuliser<br>50 mcg / mL, injection<br><br>[Originator*: Ventolin, GlaxoSmithKline]	100 mcg / dose, 200 doses, inhaler
Beclomethasone (1)	Inhaled corticosteroid (ICS)	50 mcg / dose, inhaler<br>100 mcg / dose, inhaler<br><br>[Originator*: Becotide, GlaxoSmithKline]	100 mcg / dose, 200 doses, inhaler
Budesonide (1)	Inhaled corticosteroid (ICS)	100 mcg / dose, inhaler<br>200 mcg / dose, inhaler<br><br>[Originator*: Pulmicort, AstraZeneca]	100 mcg / dose, 200 doses, inhaler
Budesonide and formoterol	Combination inhaled corticosteroid-long-acting beta-agonist (ICS-LABA)	100 + 6 mcg / dose, dry powder inhaler<br>200 + 6 mcg / dose, dry powder inhaler<br><br>[Originator*: Symbicort, AstraZeneca]	100 + 6 mcg / dose, 120 doses, inhaler<br>200 + 6 mcg / dose, 120 doses, inhaler
Tiotropium	Long-acting muscarinic antagonist (LAMA)	18 mcg / dose, capsule<br>1.25 mcg / dose, actuation<br>2.5 mcg / dose, actuation<br><br>[Originator*: Spiriva, Boehringer-Ingelheim]	18 mcg / dose, 30 doses, inhaler<br>1.25 mcg / dose, 60 doses, inhaler<br>2.5 mcg / dose, 60 doses, inhaler
Prednisolone	Corticosteroid (CS)	5 mg tablet<br>25 mg tablet<br>5 mg / mL oral liquid	40 mg / day for 5 days, oral
Ipratropium bromide	Short-acting muscarinic antagonist (SAMA)	20 mcg / dose, inhaler<br><br>[Originator*: Atrovent, Boehringer-Ingelheim]	20 mcg / dose, 200 doses, inhaler
Epinephrine / Adrenaline	Adrenaline (AD)	1mg / 1ml, injection	1 mg / ml / dose, 1 ampoule, injection

Each investigator was asked to complete the form for three facilities: one pharmacy, one HCF and CMS. Public, private, and other facilities were included and chosen using convenience sampling. Investigators visited or contacted each facility to complete the data collection form. Data for at least one facility per country had to be

Stolbrink M, *et al. Thorax* 2024; 79:676–679. doi: 10.1136/thorax-2023-221349

submitted and multiple entries were allowed. If facilities were missing, the author (MS) contacted other investigators or used publicly available data to complete the dataset.

Sampling and recruitment

Investigators were recruited through international respiratory networks and "snowballing" aiming to include as many LMICs as possible (Figure 1). LMICs were defined by 2022 World Bank category.[6]

Figure 1: Flowchart of sampling strategy for potential collaborators.

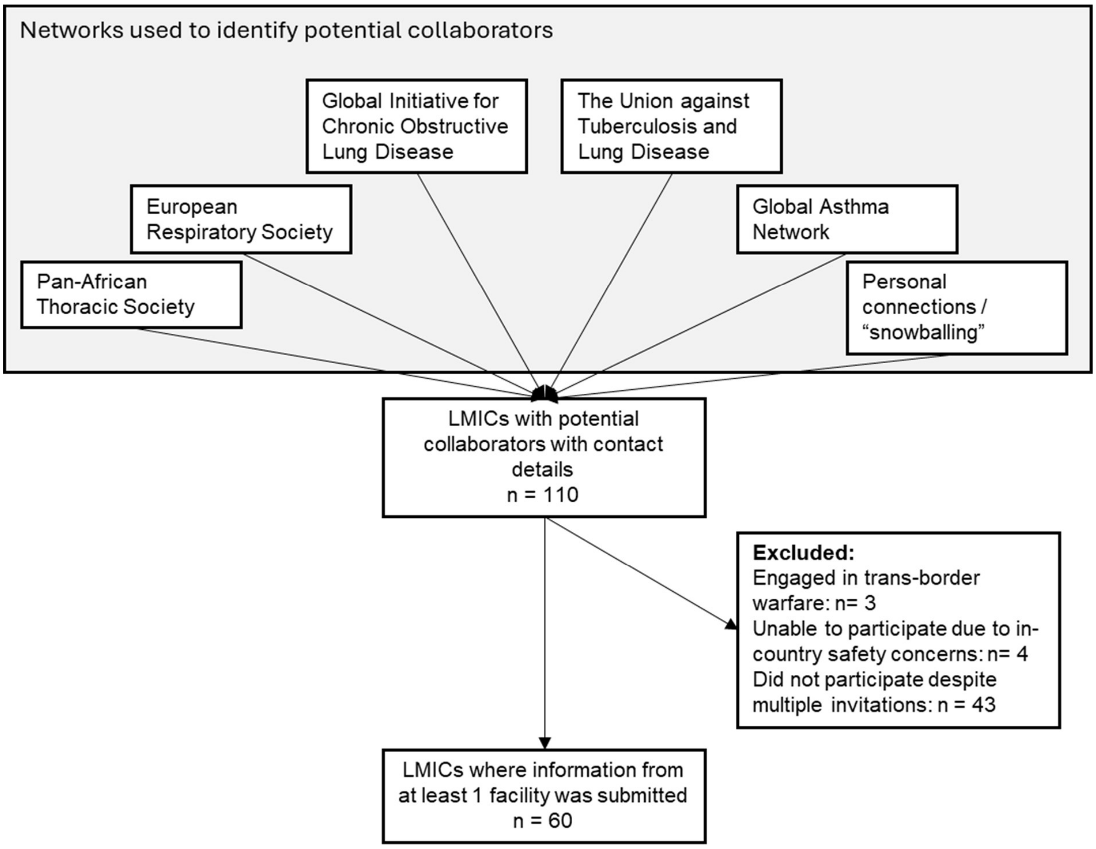

Data analysis

For each country the availability and cost of each medicine at the specified doses was reported for each of the facility types. When multiple doses were available, standardised doses were calculated which represented one month's treatment, or one course of treatment (Table 1). A medicine was available if it was present in the facility on the day of data collection. Medicine prices were expressed in local currency and converted to US$, using mean daily or monthly exchange rates.[7] A medicine was affordable if one month's treatment cost less than one day's wage of the lowest paid government worker, defined by national minimum wage defined by the International Labour Organization.[8] Affordability was only calculated for pharmacies and HCFs as CMS prices were usually wholesale prices. The costs of the cheapest products by facility were compared across countries. If there were multiple submissions for one facility the best availability and cost were presented. Descriptive analyses were applied throughout.

The funder had no role in study design, data collection, data analysis, data interpretation, or writing of the manuscript.

Appendix – Availability, cost, and affordability of essential medicines for chronic respiratory diseases in low- and middle-income countries: a cross-sectional study – Stolbrink et al.

Supplementary Table 1: Chronic Respiratory Diseases (CRDs) Medicines Survey Investigators

Country	First name	Surname	Affiliation
Albania	Eris	Mesonjesi	University Hospital Center Mother Teresa, Tirana
Algeria	Nadia	Ait-Khaled	Algeria University
Algeria	Samya	Taright	Universite d'Alger, Faculté de Medecine
Argentina	Santiago	Larrateguy	Universidad Adventista del Plata / Centro Privado de Medicina Respiratoria
Bosnia and Herzegovina	Sanela	Domuz Vujnovic	Paediatrics Clinic, University Clinical Center of Republic of Srpska
Brazil	Carolina	Barbosa Souza Santos	Programa para o Controle da Asma na Bahia - ProAR (Program for Control of Asthma in Bahia)
Burkina Faso	Abdoul Risgou	Ouédraogo	Health Training & Research Unit, Joseph KI-ZERBO University, Ouagadougou, Burkina Faso and Department of Medicine, Tengandogo University Hospital Center
Cameroon	Bertrand Hugo	Mbatchou-Ngahane	Douala General Hospital, University of Douala, Cameroon
Cameroon	Lydie	Mboumi	Pharmacie du Rocher, Douala, Cameroon
China	Yanping	Liu	Sun Yat-sen university
China	Fu-Qiang	Wen	West China Hospital, Sichuan University
China	Xi	Yan	West China Hospital, Sichuan University
China	Yutian	Zhang	West China Hospital, Sichuan University
Congo, Dem. Rep.	Patrick	Katoto	Center for Tropical Diseases and Global Health, Université Catholique de Bukavu
Congo, Dem. Rep.	Arsene Daniel	Nyalundja	Center for Tropical Diseases and Global Health, Université Catholique de Bukavu, Democratic Republic of Congo
Ecuador	Efraín	Sánchez-Angarita	Centro de Investigacion respiratorio
Egypt, Arab Rep.	Maged	Hassan	Alexandria University Faculty of Medicine
Egypt, Arab Rep.	Magda	Afifi	National Tuberculosis Control Program, Ministry of health and population
Eswatini	Willie	Siduna	University of the Western Cape
Ethiopia	Amsalu	Binegdie	College of Health Sciences,Addis Ababa University
Gambia, The	Babatunde	Awokola	Liverpool School of Tropical Medicine and Medical Research Council Unit The Gambia at London School of Hygiene and Tropical Medicine
Ghana	Rafiuk Cosmos	Yakubu	Tamale Teaching Hospital (TTH) and School of Medicine, University for Development Studies (UDS)
Guinea	Magassouba	Aboubacar Sidiki	National Tuberculosis Control Program,Conakry, Guinea
Honduras	Suyapa	Sosa	Pulmonary Medicine/Thorax National Institute
India	Sarbjeet	Khurana	IHBAS Hospital
Indonesia	Bony Wiem	Lestari	Research Center for Care and Control of Infectious Diseases (RC3ID) Universitas Padjadjaran, Bandung-Indonesia
Indonesia	Faisal	Yunus	Department of Pulmonology and Respiratory medicine, faculty of medicine universitas Indonesia - Persahabatan Hospital
Indonesia	Antonia Morita Iswari	Saktiawati	Universitas Gadjah Mada, Faculty of Medicine, Public Health, and Nursing, Department of Internal Medicine<br>Universitas Gadjah Mada, Faculty of Medicine, Public Health, and Nursing, Center for Tropical Medicine
Iran, Islamic Rep.	Mohammad Reza	Masjedi	Pulmonary Medicine, Shahid Beheshti University of Medical Sciences<br>Cancer Control Research Center, Cancer Control Foundation, Iran University of Medical Sciences, Tehran, Iran<br>Tobacco Control Research Center (TCRC), Iranian Anti-tobacco Association, Iran University of Medical Sciences, Tehran, Iran.
Iraq	Hashim	Talib Hashim	University of Warith Al-anbya, Colleges of Medicine, Karbala, Iraq
Kenya	Peter	Owiti	Stop TB Partnership / Wote Youth Development Projects
Kyrgyz Republic	Shaiirbek	Sulaimanov	Kyrgyz-Russian Slavic University and Kyrgyz State Medical Academy
Lesotho	Lawrence	Oyewusi	Partners In Health
Libya	Boshra	Abusahmin	National Centre For Disease Control
Libya	Mohamed Hadi Mohamed	Abdelhamid	National Center for Disease Control (NCDC). Biotechnology Research Center (BTRC). Tripoli-Libya.

Appendix – Availability, cost, and affordability of essential medicines for chronic respiratory diseases in low- and middle-income countries: a cross-sectional study – Stolbrink et al.

Malawi	Felix	Mkandawire	Blantyre Malaria Project
Malaysia	Ee Ming	Khoo	Department of Primary Care Medicine, Faculty of Medicine, Universiti Malaya; International Primary Care Respiratory Group
Mali	Ousmane Ibrahim	Diabate	Bamako
Mexico	Adrian	Rendon	CIPTIR, Hospital Universitario "Dr. Jose Eleuterio Gonzalez", UANL
Mexico	Berenice	Soto-Moncivais	CIPTIR, Hospital Universitario “Dr. Jose Eleuterio Gonzalez”, UANL
Mongolia	Bolyskhan	Baigabyl	Tuberculosis clinic, National Center for Communicable Diseases
Mozambique	Celso	Khosa	Instituto Nacional de Saúde- Centro de Investigação e Treino em Saúde da Polana Caniço (CISPOC), Marracuene, Mozambique
Mozambique	Cynthia	Silva	Instituto Nacional de Saúde- Centro de Investigação e Treino em Saúde da Polana Caniço (CISPOC), Marracuene, Mozambique
Nepal	Rajan	Paudel	Birat Nepal Medical Trust
Niger	Alberto	Piubello	Damien Foundation, Niamey, Niger
Niger	Kadri	Sani	Centre Hospitalier Régional, Niamey, Niger
Nigeria	Temitope	Fapohunda	Lagos State University Teaching Hospital, Nigeria
Nigeria	Olayinka	Adeyeye	Lagos State University, College of Medicine
North Macedonia	Valentina	Cvejoska Cholakovska	University Children's Clinic, Faculty of Medicine, Skopje, Ss Cyril and Methodius University of Skopje
Pakistan	Ghulam	Mustafa	College of Medicine Shaqra University Riyadh, Nishtar medical university, Multan
Peru	Javier	Cabrera-Sanchez	Facultad de Medicina, Universidad Peruana Cayetano Heredia, Lima, Peru
Romania	Diana	Deleanu	University of Medicien & Pharmacy Iuliu hatieganu
Rwanda	Jean Pierre	Sibomana	Butare University teaching hospital
Senegal	Momar	Mbodji	Dakar
Serbia	Vesna	Vekovic	Children's Hospital for Lung Diseases and Tb, University Hospital Dr Dragisa Misovic,Belgrade, Serbia
Serbia	Zorica	Zivkovic	Children's Hospital for Lung Diseases and Tb, University Hospital Dr Dragisa Misovic, Belgrade, Serbia<br>Faculty of Pharmacy in Novi Sad, University Business Academy Novi Sad, Serbia
Somalia	Osman Muhyadin	Abdulle	Forlanini Hospital,National Tuberculosis Program and Somaville University,,Mogadishu, Somalia
South Sudan	Babiker	Adam	M-Pharma co Ltd
Sri Lanka	Sisira	Siribaddana	Rajarata University of Sri Lanka, Teaching Hospital Anuradhapura
Sudan	Rana	Ahmed	The Epidemiological Laboratory, Khartoum, Sudan
Sudan	Mahdia	Elhadi	University of Hail
Sudan	Mohamed	Elmustafa	University of Gezira; Wad Medani College of Medical Sciences and Technology
Syrian Arab Republic	Yousser	Mohammad	Tishreen University, Latakia; Al Sham Private University, Damascus, Syria
Tanzania	Stellah	Mpagama	Kibong'oto Infectious Diseases Hospital
Tanzania	Bibie	Said	Kibong'oto Infectious Diseases Hospital
Thailand	Mongkol	Lao-Araya	Chiang Mai University Hospital, Faculty of Medicine, Chiang Mai University
Timor-Leste	Benilda Trias	de Gula	Saint Paul Clinic of the Sisters of St. Paul of Chartres Congregation
Tunisia	Agnes	Hamzaoui	Hopital A Mami, Ariana and Medicine School, Tunis
Turkiye	Kübra	Tunçel	University of Gazi
Turkiye	Islam	Sangac	
Turkiye	Tuğçe	Tayyar	Lavanta Pharmacy
Turkiye	Aygün	Gürgöze	Polatlı Can Hospital
Uganda	Rebecca	Nantanda	Makerere University Ung Institute, College of Health Sciences, Makerere University
Venezuela	Maria	Montes de Oca	Universidad Central de Venezuela, Centro Medico de Caracas
Venezuela	Juan	Catari	Centro Medico de Caracas
Vietnam	Tran Thien Quan	Vu	University of Medicine and Pharmacy at Ho Chi Minh city

Stolbrink M, *et al. Thorax* 2024; 79:676–679. doi: 10.1136/thorax-2023-221349

Appendix – Availability, cost, and affordability of essential medicines for chronic respiratory diseases in low- and middle-income countries: a cross-sectional study – Stolbrink et al.

Yemen, Rep.	Mohammed	Mohammed	Al Taaon pharmacy
Yemen, Rep.	Ruba	Khaled	
Yemen, Rep.	Weiam	Hussein	
Zambia	Charles	Mataya Mphuka	Livingstone Central Hospital
Zimbabwe	Terrence	Rudado Musekiwa	Chimhanda District Hospital

Appendix – Availability, cost, and affordability of essential medicines for chronic respiratory diseases in low- and middle-income countries: a cross-sectional study – Stolbrink et al.

Supplementary Table 2: STROBE Statement[1]

	Item No.	Recommendation	Page No.
**Title and abstract**	1	(*a*) Indicate the study's design with a commonly used term in the title or the abstract	1
		(*b*) Provide in the abstract an informative and balanced summary of what was done and what was found	3
**Introduction**			
Background/rationale	2	Explain the scientific background and rationale for the investigation being reported	4
Objectives	3	State specific objectives, including any prespecified hypotheses	4
**Methods**			
Study design	4	Present key elements of study design early in the paper	4, Appendix
Setting	5	Describe the setting, locations, and relevant dates, including periods of recruitment, exposure, follow-up, and data collection	4, Appendix
Participants	6	(*a*) *Cohort study*—Give the eligibility criteria, and the sources and methods of selection of participants. Describe methods of follow-up<br>*Case-control study*—Give the eligibility criteria, and the sources and methods of case ascertainment and control selection. Give the rationale for the choice of cases and controls<br>*Cross-sectional study*—Give the eligibility criteria, and the sources and methods of selection of participants	4, Appendix
		(*b*) *Cohort study*—For matched studies, give matching criteria and number of exposed and unexposed<br>*Case-control study*—For matched studies, give matching criteria and the number of controls per case	N/A
Variables	7	Clearly define all outcomes, exposures, predictors, potential confounders, and effect modifiers. Give diagnostic criteria, if applicable	4, Appendix
Data sources/ measurement	8*	For each variable of interest, give sources of data and details of methods of assessment (measurement). Describe comparability of assessment methods if there is more than one group	4, Appendix
Bias	9	Describe any efforts to address potential sources of bias	4, Appendix
Study size	10	Explain how the study size was arrived at	4, Appendix
Quantitative variables	11	Explain how quantitative variables were handled in the analyses. If applicable, describe which groupings were chosen and why	4, Appendix
Statistical methods	12	(*a*) Describe all statistical methods, including those used to control for confounding	4, Appendix
		(*b*) Describe any methods used to examine subgroups and interactions	4, Appendix
		(*c*) Explain how missing data were addressed	4, Appendix
		(*d*) *Cohort study*—If applicable, explain how loss to follow-up was addressed<br>*Case-control study*—If applicable, explain how matching of cases and controls was addressed<br>*Cross-sectional study*—If applicable, describe analytical methods taking account of sampling strategy	N/A
		(*e*) Describe any sensitivity analyses	N/A
Participants	13*	(a) Report numbers of individuals at each stage of study—eg numbers potentially eligible, examined for eligibility, confirmed eligible, included in the study, completing follow-up, and analysed	4
		(b) Give reasons for non-participation at each stage	N/A
		(c) Consider use of a flow diagram	N/A
Descriptive data	14*	(a) Give characteristics of study participants (eg demographic, clinical, social) and information on exposures and potential confounders	4, Appendix
		(b) Indicate number of participants with missing data for each variable of interest	Appendix
		(c) *Cohort study*—Summarise follow-up time (eg, average and total amount)	N/A
Outcome data	15*	*Cohort study*—Report numbers of outcome events or summary measures over time	
		Case-control study—Report numbers in each exposure category, or summary measures of exposure	

Stolbrink M, *et al. Thorax* 2024; 79:676–679. doi: 10.1136/thorax-2023-221349

Appendix – Availability, cost, and affordability of essential medicines for chronic respiratory diseases in low- and middle-income countries: a cross-sectional study – Stolbrink et al.

		Cross-sectional study—Report numbers of outcome events or summary measures	4-7, Appendix
Main results	16	(*a*) Give unadjusted estimates and, if applicable, confounder-adjusted estimates and their precision (eg, 95% confidence interval). Make clear which confounders were adjusted for and why they were included	4-7, Appendix
		(*b*) Report category boundaries when continuous variables were categorized	4-7, Appendix
		(*c*) If relevant, consider translating estimates of relative risk into absolute risk for a meaningful time period	N/A
Other analyses	17	Report other analyses done—eg analyses of subgroups and interactions, and sensitivity analyses	4-7, Appendix
Key results	18	Summarise key results with reference to study objectives	8
Limitations	19	Discuss limitations of the study, taking into account sources of potential bias or imprecision. Discuss both direction and magnitude of any potential bias	8
Interpretation	20	Give a cautious overall interpretation of results considering objectives, limitations, multiplicity of analyses, results from similar studies, and other relevant evidence	8
Generalisability	21	Discuss the generalisability (external validity) of the study results	8
**Other information**			
Funding	22	Give the source of funding and the role of the funders for the present study and, if applicable, for the original study on which the present article is based	9

Information on the STROBE Initiative is available at www.strobe-statement.org.

Appendix – Availability, cost, and affordability of essential medicines for chronic respiratory diseases in low- and middle-income countries: a cross-sectional study – Stolbrink et al.

Supplementary Table 3: Overview of included LMICs and facilities by income group and WHO region

LMIC: Low-income and middle-income country; WHO: World Health Organization; HCF: Healthcare facility; CMS: Central medicine stores; No information: NIA available; NGO: Non-governmental organisation.

(1) Data for 3 private pharmacies submitted; (2) Data for 2 private pharmacies submitted; (3) Data for 2 private pharmacies submitted; (4) Data for 3 HCFs submitted, 2 public and 1 private HCF; (5) Data for 2 public HCFs submitted; (6) Data for 3 pharmacies submitted, 2 pubic and 1 private pharmacy.

Country	World Bank Income Group[6]	WHO Region[9]	Pharmacy – type of facility	HCF – type of facility	CMS – type of facility
Albania	Upper middle income	Europe	Private	Public	NIA
Algeria	Lower middle income	Africa	Private	Public	CMS
Angola	Lower middle income	Africa	Private	Public	CMS
Argentina	Upper middle income	Americas	Private	Public	CMS
Bosnia and Herzegovina	Upper middle income	Europe	Private	Public	NIA
Brazil	Upper middle income	Americas	Private	Public	CMS
Burkina Faso	Low income	Africa	Private	Public	NIA
Cameroon	Lower middle income	Africa	Private	Public	CMS
Chad	Low income	Africa	NIA	NIA	CMS
China	Upper middle income	Western Pacific	Private (1)	Public	CMS
Democratic Republic of Congo	Low income	Africa	Private	Public	CMS
Ecuador	Upper middle income	Americas	Private	Private	CMS
Egypt	Lower middle income	Eastern Mediterranean	Private (2)	Private	CMS
Eswatini	Lower middle income	Africa	Private	NIA	CMS
Ethiopia	Low income	Africa	Private	Public	CMS
Ghana	Lower middle income	Africa	Private	Public	CMS
Guinea	Low income	Africa	Private	NIA	CMS
Honduras	Lower middle income	Americas	Private	Public	NIA
India	Lower middle income	South-East Asia	Private	Public	CMS
Indonesia	Lower middle income	South-East Asia	Private (3)	Private & Public (4)	CMS
Iran	Lower middle income	Eastern Mediterranean	Private	Public	CMS
Iraq	Upper middle income	Eastern Mediterranean	Private	Public	NIA
Kenya	Lower middle income	Africa	Private	Public	NIA
Kyrgyzstan	Lower middle income	Europe	Private	Public	CMS
Lesotho	Lower middle income	Africa	NIA	NGO	CMS
Libya	Upper middle income	Eastern Mediterranean	Private	Public	CMS
Malawi	Low income	Africa	Private	Public (5)	CMS
Malaysia	Upper middle income	Western Pacific	Private	Public	CMS
Mali	Low income	Africa	Private	Public	CMS
Mexico	Upper middle income	Americas	Private	Private	NIA
Moçambique	Low income	Africa	Private	Public	CMS
Mongolia	Lower middle income	Western Pacific	Private & Public (6)	Public	CMS
Nepal	Lower middle income	South-East Asia	Private	Public	CMS
Niger	Low income	Africa	Private	Public	CMS
Nigeria	Lower middle income	Africa	Private	Public	CMS
North Macedonia	Upper middle income	Europe	Public	Public	CMS

Stolbrink M, *et al. Thorax* 2024; 79:676–679. doi: 10.1136/thorax-2023-221349

Appendix – Availability, cost, and affordability of essential medicines for chronic respiratory diseases in low- and middle-income countries: a cross-sectional study – Stolbrink et al.

Pakistan	Lower middle income	Eastern Mediterranean	Public	Public	CMS
Peru	Upper middle income	Americas	Private	Public	CMS
Romania	Upper middle income	Europe	Private	Public	CMS
Rwanda	Low income	Africa	Private	Public	NIA
Senegal	Lower middle income	Africa	Private	Public	CMS
Serbia	Upper middle income	Europe	Private	Public	CMS
Somalia	Low income	Eastern Mediterranean	Private	Private	NIA
South Africa	Upper middle income	Africa	Private	Public	CMS
South Sudan	Low income	Africa	Private	NIA	NIA
Sri Lanka	Lower middle income	South-East Asia	Private	Public	CMS
Sudan	Low income	Eastern Mediterranean	Private	Public	CMS
Syria	Low income	Eastern Mediterranean	Private	Public	CMS
Tanzania	Lower middle income	Africa	Private	Public	CMS
Thailand	Upper middle income	South-East Asia	Public	Public	NIA
The Gambia	Low income	Africa	Private	Public	CMS
Timor Leste	Lower middle income	South-East Asia	NIA	Private	NIA
Tunisia	Lower middle income	Eastern Mediterranean	Public	Public	CMS
Turkiye	Upper middle income	Europe	Private	Private	CMS
Uganda	Low income	Africa	Private	NGO	CMS
Venezuela		Americas	Private	Private	NIA
Viet Nam	Lower middle income	Western Pacific	Private	Public	CMS
Yemen	Low income	Eastern Mediterranean	Public	Private	NIA
Zambia	Low income	Africa	Private	Public	CMS
Zimbabwe	Lower middle income	Africa	Private	Public	CMS

Stolbrink M, *et al. Thorax* 2024; 79:676–679. doi: 10.1136/thorax-2023-221349

Appendix – Availability, cost, and affordability of essential medicines for chronic respiratory diseases in low- and middle-income countries: a cross-sectional study – Stolbrink et al.

Supplementary Table 4: Availability, cost, and affordability of SABA by country and type of facility.

Cost is for standardised dose and formulation (inhaled salbutamol 100mcg/dose, 200 doses). CMS costs are wholesale costs, unsuitable for affordability calculations. Affordable: one month's treatment costs less than one day's wage of national minimum wage. $: US$; SABA: Short-acting beta-agonist inhaler; HCF: Healthcare facility; CMS: Central medicine stores; NIA: No information available; DRC: Democratic Republic of Congo; NGO: Non-governmental organisation.

Country	Minimum daily wage ($)	Pharmacy				HCF				CMS	
		Type of facility	SABA available?	SABA cost ($)	SABA affordable?	Type of facility	SABA available?	SABA cost ($)	SABA affordable?	SABA available?	SABA cost ($)
Albania	11.09	Private	Yes	0.00	Yes	Public	Yes	1.56	Yes	NIA	NIA
Algeria	5.39	Private	Yes	0.00	Yes	Public	Unavailable	Unavailable	Unavailable	Unavailable	Unavailable
Angola	2.46	Private	Yes	4.96	Unaffordable	Public	Unavailable	Unavailable	Unavailable	Yes	1.39
Argentina	15.60	Private	Yes	9.40	Yes	Public	Yes	2.94	Yes	Yes	5.60
Bosnia and Herzegovina	10.89	Private	Yes	2.61	Yes	Public	Yes	2.70	Yes	NIA	NIA
Brazil	8.86	Private	Yes	0.00	Yes	Public	Yes	0.00	Yes	Unavailable	Unavailable
Burkina Faso	2.13	Private	Yes	3.68	Unaffordable	Public	Unavailable	Unavailable	Unavailable	NIA	NIA
Cameroon	2.23	Private	Yes	3.20	Unaffordable	Public	Yes	4.16	Unaffordable	Yes	1.33
Chad	3.70	NIA	NIA	NIA	NIA	NIA	NIA	NIA	NIA	Yes	NIA
China	10.62	Private	Yes	3.40	Yes	Public	Unavailable	Unavailable	Unavailable	Yes	2.83
DRC	3.54	Private	Yes	4.00	Unaffordable	Public	Yes	10.50	Unaffordable	Yes	3.00
Ecuador	15.17	Private	Yes	7.80	Yes	Private	Yes	5.65	Yes	Yes	1.05
Egypt	5.85	Private	Yes	0.61	Yes	Private	Yes	1.09	Yes	Yes	0.59
Eswatini	0.92	Private	Yes	2.72	Unaffordable	NIA	NIA	NIA	NIA	Yes	1.39
Ethiopia	0.31	Private	Yes	3.78	Unaffordable	Public	Yes	3.34	Unaffordable	Yes	NIA
Ghana	1.19	Private	Yes	3.50	Unaffordable	Public	Yes	2.28	Unaffordable	Yes	1.49
Guinea	2.44	Private	Unavailable	Unavailable	Unavailable	NIA	NIA	NIA	NIA	Unavailable	Unavailable
Honduras	14.82	Private	Yes	4.45	Yes	Public	Yes	4.12	Yes	NIA	NIA
India	2.14	Private	Yes	1.88	Yes	Public	Yes	0.65	Yes	Yes	0.66
Indonesia	7.22	Private	Yes	11.31	Unaffordable	Private & Public	Yes	6.44	Yes	Yes	1.85
Iran	20.44	Private	Yes	2.20	Yes	Public	Yes	2.20	Yes	Yes	2.20

Stolbrink M, *et al. Thorax* 2024; 79:676–679. doi: 10.1136/thorax-2023-221349

Appendix – Availability, cost, and affordability of essential medicines for chronic respiratory diseases in low- and middle-income countries: a cross-sectional study – Stolbrink et al.

Iraq	9.43	Private	Yes	4.20	Yes	Public	Yes	3.50	Yes	NIA	NIA
Kenya	5.25	Private	Yes	2.55	Yes	Public	Yes	2.98	Yes	NIA	NIA
Kyrgyzstan	0.88	Private	Unavailable	Unavailable	Unavailable	Public	Yes	2.59	Unaffordable	Yes	1.62
Lesotho	3.60	NIA	NIA	NIA	NIA	NGO	Yes	11.54	Unaffordable	Yes	NIA
Libya	3.59	Private	Yes	2.90	Yes	Public	Unavailable	Unavailable	Unavailable	Unavailable	Unavailable
Malawi	1.92	Private	Unavailable	Unavailable	Unavailable	Public	Yes	0.00	Yes	Yes	1.35
Malaysia	12.88	Private	Yes	2.55	Yes	Public	Yes	1.78	Yes	Yes	2.98
Mali	2.46	Private	Yes	7.68	Unaffordable	Public	Unavailable	Unavailable	Unavailable	Unavailable	Unavailable
Mexico	15.07	Private	Yes	3.10	Yes	Private	Yes	14.95	Yes	NIA	NIA
Moçambique	4.80	Private	Yes	5.57	Unaffordable	Public	Unavailable	Unavailable	Unavailable	Unavailable	Unavailable
Mongolia	4.85	Private & Public	Yes	1.00	Yes	Public	Yes	3.15	Yes	Unavailable	Unavailable
Nepal	4.56	Private	Yes	1.74	Yes	Public	Yes	1.62	Yes	Yes	0.00
Niger	2.31	Private	Yes	5.22	Unaffordable	Public	Unavailable	Unavailable	Unavailable	Yes	3.51
Nigeria	2.77	Private	Yes	3.12	Unaffordable	Public	Yes	4.08	Unaffordable	Yes	3.01
North Macedonia	17.37	Public	Yes	2.65	Yes	Public	Unavailable	Unavailable	Unavailable	Yes	2.64
Pakistan	4.23	Public	Yes	0.00	Yes	Public	Yes	0.88	Yes	Yes	1.21
Peru	9.90	Private	Yes	1.73	Yes	Public	Yes	0.96	Yes	Yes	1.26
Romania	20.32	Private	Yes	2.03	Yes	Public	Yes	2.24	Yes	Yes	NIA
Rwanda	0.09	Private	Yes	2.28	Unaffordable	Public	Yes	2.28	Unaffordable	NIA	NIA
Senegal	3.34	Private	Yes	2.95	Yes	Public	Unavailable	Unavailable	Unavailable	Yes	2.12
Serbia	16.35	Private	Yes	NIA	NIA	Public	Yes	1.92	Yes	Yes	NIA
Somalia	0.10	Private	Yes	5.14	Unaffordable	Private	Unavailable	Unavailable	Unavailable	NIA	NIA
South Africa	9.37	Private	Yes	2.82	Yes	Public	Yes	0.96	Yes	Yes	1.00
South Sudan	5.05	Private	Yes	6.00	Unaffordable	NIA	NIA	NIA	NIA	NIA	NIA
Sri Lanka	1.35	Private	Yes	2.05	Unaffordable	Public	Yes	0.00	Yes	Yes	0.36
Sudan	0.03	Private	Yes	2.40	Unaffordable	Public	Yes	1.32	Unaffordable	Unavailable	Unavailable
Syria	0.55	Private	Yes	0.90	Unaffordable	Public	Yes	0.00	Yes	Yes	0.77
Tanzania	2.18	Private	Yes	4.00	Unaffordable	Public	Yes	1.80	Yes	Yes	1.20
Thailand	9.05	Public	Yes	5.27	Yes	Public	Yes	1.40	Yes	NIA	NIA

Appendix – Availability, cost, and affordability of essential medicines for chronic respiratory diseases in low- and middle-income countries: a cross-sectional study – Stolbrink et al.

The Gambia	0.91	Private	Yes	8.19	Unaffordable	Public	Yes	0.00	Yes	Yes	3.46
Timor Leste	4.43	NIA	NIA	NIA	NIA	Private	Yes	25.00	Unaffordable	NIA	NIA
Tunisia	4.74	Public	Yes	1.94	Yes	Public	Yes	1.95	Yes	Yes	NIA
Turkiye	13.90	Private	Yes	1.94	Yes	Private	Yes	2.40	Yes	Yes	0.30
Uganda	0.07	Private	Yes	NIA	NIA	NGO	Yes	3.00	Unaffordable	Yes	NIA
Venezuela	0.94	Private	Yes	8.05	Unaffordable	Private	Yes	10.18	Unaffordable	NIA	NIA
Viet Nam	6.00	Private	Unavailable	Unavailable	Unavailable	Public	Yes	3.20	Yes	Unavailable	Unavailable
Yemen	3.23	Public	Yes	0.06	Yes	Private	Yes	8.79	Unaffordable	NIA	NIA
Zambia	2.59	Private	Yes	4.99	Unaffordable	Public	Unavailable	Unavailable	Unavailable	Unavailable	Unavailable
Zimbabwe	11.58	Private	Yes	9.45	Yes	Public	Yes	3.78	Yes	Yes	5.40

Stolbrink M, *et al. Thorax* 2024; 79:676–679. doi: 10.1136/thorax-2023-221349

Appendix – Availability, cost, and affordability of essential medicines for chronic respiratory diseases in low- and middle-income countries: a cross-sectional study – Stolbrink et al.

Supplementary Table 5: Availability, cost, and affordability of ICS by country and type of facility.

Cost is for standardised dose and formulation (inhaled beclomethasone or budesonide 100 mcg/dose, 200 doses). CMS costs are wholesale costs, unsuitable for affordability calculations. Affordable: one month's treatment costs less than one day's wage of national minimum wage. $: US$; ICS: inhaled corticosteroid; HCF: Healthcare facility; CMS: Central medicine stores; NIA: No information available; DRC: Democratic Republic of Congo; NGO: Non-governmental organisation.

(1): Clenil 250mcg/dose available in pharmacy; (2): Flixotide 50 mcg/dose, 125 cmg/dose, 250 mcg/dose available in pharmacy and HCF; (3): Oxalair (fluticasone) 125 mcg/dose available in pharmacy; (4): Flixotide 125 mcg/dose available in pharmacy; (5) ciclesonide 160mcg/dose available in HCF; (6): Flixotide 50 mcg/dose available in pharmacy, HCF, CMS; (7): Flixotide 50mcg/dose, 125 mcg/dose available in CMS; (8): Mometasone 50 mcg/dose available in pharmacy; (9): Fluticasone 100 mcg/dose, 125 mcg/dose available in pharmacy and HCF.

Country	Minimum daily wage ($)	Pharmacy				HCF				CMS	
		Type of facility	ICS available?	ICS cost ($)	ICS affordable?	Type of facility	ICS available?	ICS cost ($)	ICS affordable?	ICS available?	ICS cost ($)
Albania	11.09	Private	Yes	0.00	Yes	Public	Yes	2.87	Yes	NIA	NIA
Algeria	5.39	Private	Unavailable (1)	Unavailable	Unavailable	Public	Unavailable	Unavailable	Unavailable	Unavailable	Unavailable
Angola	2.46	Private	Unavailable	Unavailable	Unavailable	Public	Unavailable	Unavailable	Unavailable	Unavailable	Unavailable
Argentina	15.60	Private	Yes	6.41	Yes	Public	Yes	1.95	Yes	Yes	5.95
Bosnia and Herzegovina	10.89	Private	Unavailable (2)	Unavailable	Unavailable	Public	Unavailable (2)	Unavailable	Unavailable	NIA	NIA
Brazil	8.86	Private	Yes	0.00	Yes	Public	Yes	0.00	Yes	Yes	0.00
Burkina Faso	2.13	Private	Unavailable (3)	Unavailable	Unavailable	Public	Unavailable	Unavailable	Unavailable	NIA	NIA
Cameroon	2.23	Private	Yes	19.20	Unaffordable	Public	Unavailable	Unavailable	Unavailable	Unavailable	Unavailable
Chad	3.70	NIA	NIA	NIA	NIA	NIA	NIA	NIA	NIA	Unavailable	Unavailable
China	10.62	Private	Unavailable	Unavailable	Unavailable	Public	Unavailable	Unavailable	Unavailable	Unavailable	Unavailable
DRC	3.54	Private	Yes	12.00	Unaffordable	Public	Yes	5.25	Unaffordable	Unavailable	Unavailable
Ecuador	15.17	Private	Yes	5.50	Yes	Private	Yes	7.00	Yes	Unavailable	Unavailable
Egypt	5.85	Private	Yes	1.39	Yes	Private	Yes	2.79	Yes	Yes	1.31
Eswatini	0.92	Private	Yes	8.11	Unaffordable	NIA	NIA	NIA	NIA	Yes	2.77
Ethiopia	0.31	Private	Unavailable	Unavailable	Unavailable	Public	Yes	3.01	Unaffordable	Yes	0.00
Ghana	1.19	Private	Unavailable	Unavailable	Unavailable	Public	Unavailable	Unavailable	Unavailable	Unavailable	Unavailable
Guinea	2.44	Private	Unavailable	Unavailable	Unavailable	NIA	NIA	NIA	NIA	Unavailable	Unavailable

Stolbrink M, *et al. Thorax* 2024; 79:676–679. doi: 10.1136/thorax-2023-221349

Appendix – Availability, cost, and affordability of essential medicines for chronic respiratory diseases in low- and middle-income countries: a cross-sectional study – Stolbrink et al.

Honduras	14.82	Private	Yes	6.24	Yes	Public	Yes	14.42	Yes	NIA	NIA
India	2.14	Private	Yes	2.24	Unaffordable	Public	Yes	1.00	Yes	Yes	1.00
Indonesia	7.22	Private	Yes	19.81	Unaffordable	Private & Public	Yes	9.92	Unaffordable	Unavailable	Unavailable
Iran	20.44	Private	Yes	1.51	Yes	Public	Unavailable	Unavailable	Unavailable	Yes	1.51
Iraq	9.43	Private	Unavailable	Unavailable	Unavailable	Public	Unavailable	Unavailable	Unavailable	NIA	NIA
Kenya	5.25	Private	Yes	6.38	Unaffordable	Public	Unavailable	Unavailable	Unavailable	NIA	NIA
Kyrgyzstan	0.88	Private	Yes	4.62	Unaffordable	Public	Yes	3.58	Unaffordable	Yes	4.62
Lesotho	3.60	NIA	NIA	NIA	NIA	NGO	Yes	5.77	Unaffordable	Yes	0.00
Libya	3.59	Private	Unavailable (4)	Unavailable	Unavailable	Public	Unavailable	Unavailable	Unavailable	Unavailable	Unavailable
Malawi	1.92	Private	Unavailable	Unavailable	Unavailable	Public	Yes	0.00	Yes	Yes	7.28
Malaysia	12.88	Private	Yes	5.75	Yes	Public	Unavailable (5)	Unavailable	Unavailable	Yes	5.46
Mali	2.46	Private	Unavailable	Unavailable	Unavailable	Public	Unavailable	Unavailable	Unavailable	Unavailable	Unavailable
Mexico	15.07	Private	Yes	9.10	Yes	Private	Yes	49.65	Unaffordable	NIA	NIA
Moçambique	4.80	Private	Unavailable	Unavailable	Unavailable	Public	Yes	0.08	Yes	Yes	0.00
Mongolia	4.85	Private & Public	Unavailable	Unavailable	Unavailable	Public	Yes	9.30	Unaffordable	Unavailable	Unavailable
Nepal	4.56	Private	Unavailable	Unavailable	Unavailable	Public	Yes	2.25	Yes	Unavailable	Unavailable
Niger	2.31	Private	Yes	30.68	Unaffordable	Public	Unavailable	Unavailable	Unavailable	Unavailable	Unavailable
Nigeria	2.77	Private	Unavailable (6)	Unavailable	Unavailable	Public	Unavailable (6)	Unavailable	Unavailable	Unavailable (6)	Unavailable
North Macedonia	17.37	Public	Unavailable	Unavailable	Unavailable	Public	Unavailable	Unavailable	Unavailable	Unavailable	Unavailable (7)
Pakistan	4.23	Public	Yes	0.00	Yes	Public	Yes	1.98	Yes	Yes	0.45
Peru	9.90	Private	Yes	5.96	Yes	Public	Yes	1.01	Yes	Yes	2.51
Romania	20.32	Private	Unavailable	Unavailable	Unavailable	Public	Yes	3.26	Yes	Unavailable	Unavailable
Rwanda	0.09	Private	Unavailable (8)	Unavailable	Unavailable	Public	Unavailable	Unavailable	Unavailable	NIA	NIA
Senegal	3.34	Private	Yes	11.94	Unaffordable	Public	Unavailable	Unavailable	Unavailable	Unavailable	Unavailable
Serbia	16.35	Private	Yes	0.00	Yes	Public	Yes	3.16	Yes	Yes	0.00
Somalia	0.10	Private	Unavailable	Unavailable	Unavailable	Private	Yes	7.20	Unaffordable	NIA	NIA
South Africa	9.37	Private	Yes	2.16	Yes	Public	Yes	2.28	Yes	Yes	2.41

Appendix – Availability, cost, and affordability of essential medicines for chronic respiratory diseases in low- and middle-income countries: a cross-sectional study – Stolbrink et al.

South Sudan	5.05	Private	Yes	3.00	Yes	NIA	NIA	NIA	NIA	NIA	NIA
Sri Lanka	1.35	Private	Unavailable	Unavailable	Unavailable	Public	Unavailable	Unavailable	Unavailable	Yes	0.57
Sudan	0.03	Private	Yes	5.00	Unaffordable	Public	Unavailable	Unavailable	Unavailable	Unavailable	Unavailable
Syria	0.55	Private	Yes	0.54	Yes	Public	Yes	0.00	Yes	Yes	0.46
Tanzania	2.18	Private	Yes	5.40	Unaffordable	Public	Yes	5.00	Unaffordable	Yes	3.40
Thailand	9.05	Public	Unavailable	Unavailable	Unavailable	Public	Yes	1.40	Yes	NIA	NIA
The Gambia	0.91	Private	Yes	14.56	Unaffordable	Public	Unavailable	Unavailable	Unavailable	Unavailable	Unavailable
Timor Leste	4.43	NIA	NIA	NIA	NIA	Private	Unavailable	Unavailable	Unavailable	NIA	NIA
Tunisia	4.74	Public	Yes	2.08	Yes	Public	Yes	0.00	Yes	Yes	0.00
Turkiye	13.90	Private	Yes	3.52	Yes	Private	Yes	3.52	Yes	Yes	0.69
Uganda	0.07	Private	Unavailable	Unavailable	Unavailable	NGO	Yes	6.00	Unaffordable	Unavailable	Unavailable
Venezuela	0.94	Private	Yes	5.34	Unaffordable	Private	Yes	9.34	Unaffordable	NIA	NIA
Viet Nam	6.00	Private	Unavailable (9)	Unavailable	Unavailable	Public	Unavailable (9)	Unavailable	Unavailable	Unavailable	Unavailable
Yemen	3.23	Public	Unavailable	Unavailable	Unavailable	Private	Yes	0.00	Yes	NIA	NIA
Zambia	2.59	Private	Unavailable	Unavailable	Unavailable	Public	Unavailable	Unavailable	Unavailable	Unavailable	Unavailable
Zimbabwe	11.58	Private	Yes	18.90	Unaffordable	Public	Unavailable	Unavailable	Unavailable	Yes	15.12

Stolbrink M, *et al. Thorax* 2024; 79:676–679. doi: 10.1136/thorax-2023-221349

Appendix – Availability, cost, and affordability of essential medicines for chronic respiratory diseases in low- and middle-income countries: a cross-sectional study – Stolbrink et al.

Supplementary Table 6: Availability, cost, and affordability of ICS-LABA (200+6mcg/dose) by country and type of facility.

Cost is for standardised dose and formulation (inhaled budesonide-formoterol or beclomethasone-formoterol combination 200+6 mcg/dose, 120 doses). CMS costs are wholesale costs, unsuitable for affordability calculations. Affordable: one month's treatment costs less than one day's wage of national minimum wage. $: US$; ICS-LABA: inhaled corticosteroid-long-acting beta-agonist combination; HCF: Healthcare facility; CMS: Central medicine stores; NIA: No information available; DRC: Democratic Republic of Congo; NGO: Non-governmental organisation.

Country	Minimum daily wage ($)	Pharmacy				HCF				CMS	
		Type of facility	ICS-LABA (200+6) available?	ICS-LABA (200+6) cost ($)	ICS-LABA (200+6) affordable?	Type of facility	ICS-LABA (200+6) available?	ICS-LABA (200+6) cost ($)	ICS-LABA (200+6) affordable?	ICS-LABA (200+6) available?	ICS-LABA (200+6) cost ($)
Albania	11.09	Private	Yes	0.00	Yes	Public	Yes	28.18	Unaffordable	NIA	NIA
Algeria	5.39	Private	Unavailable	Unavailable	Unavailable	Public	Unavailable	Unavailable	Unavailable	Unavailable	Unavailable
Angola	2.46	Private	Unavailable	Unavailable	Unavailable	Public	Unavailable	Unavailable	Unavailable	Unavailable	Unavailable
Argentina	15.60	Private	Yes	72.80	Unaffordable	Public	Unavailable	Unavailable	Unavailable	Yes	19.60
Bosnia and Herzegovina	10.89	Private	Unavailable	Unavailable	Unavailable	Public	Yes	79.40	Unaffordable	NIA	NIA
Brazil	8.86	Private	Yes	26.23	Unaffordable	Public	Unavailable	Unavailable	Unavailable	Yes	0.00
Burkina Faso	2.13	Private	Yes	25.96	Unaffordable	Public	Unavailable	Unavailable	Unavailable	NIA	NIA
Cameroon	2.23	Private	Yes	19.20	Unaffordable	Public	Yes	23.92	Unaffordable	Unavailable	Unavailable
Chad	3.70	Unavailable	NIA	NIA	NIA	Unavailable	NIA	NIA	NIA	Unavailable	Unavailable
China	10.62	Private	Unavailable	Unavailable	Unavailable	Public	Unavailable	Unavailable	Unavailable	Unavailable	Unavailable
DRC	3.54	Private	Yes	15.00	Unaffordable	Public	Yes	16.00	Unaffordable	Unavailable	Unavailable
Ecuador	15.17	Private	Yes	12.56	Yes	Private	Yes	16.20	Unaffordable	Unavailable	Unavailable
Egypt	5.85	Private	Yes	5.60	Yes	Private	Unavailable	Unavailable	Unavailable	Yes	6.74
Eswatini	0.92	Private	Unavailable	Unavailable	Unavailable	Unavailable	NIA	NIA	NIA	Unavailable	Unavailable
Ethiopia	0.31	Private	Unavailable	Unavailable	Unavailable	Public	Unavailable	Unavailable	Unavailable	Unavailable	Unavailable
Ghana	1.19	Private	Unavailable	Unavailable	Unavailable	Public	Yes	10.59	Unaffordable	Yes	7.86
Guinea	2.44	Private	Unavailable	Unavailable	Unavailable	Unavailable	NIA	NIA	NIA	Unavailable	Unavailable
Honduras	14.82	Private	Yes	60.18	Unaffordable	Public	Yes	57.68	Unaffordable	NIA	NIA
India	2.14	Private	Yes	4.22	Unaffordable	Public	Unavailable	Unavailable	Unavailable	Unavailable	Unavailable
Indonesia	7.22	Private	Unavailable	Unavailable	Unavailable	Public	Yes	22.40	Unaffordable	Unavailable	Unavailable

Stolbrink M, *et al. Thorax* 2024; 79:676–679. doi: 10.1136/thorax-2023-221349

Appendix – Availability, cost, and affordability of essential medicines for chronic respiratory diseases in low- and middle-income countries: a cross-sectional study – Stolbrink et al.

Iran	20.44	Private	Yes	8.40	Yes	Public	Unavailable	Unavailable	Unavailable	Yes	8.40
Iraq	9.43	Private	Unavailable	Unavailable	Unavailable	Public	Unavailable	Unavailable	Unavailable	NIA	NIA
Kenya	5.25	Private	Unavailable	Unavailable	Unavailable	Public	Unavailable	Unavailable	Unavailable	NIA	NIA
Kyrgyzstan	0.88	Private	Unavailable	Unavailable	Unavailable	Public	Yes	20.62	Unaffordable	Unavailable	Unavailable
Lesotho	3.60	NIA	NIA	NIA	NIA	NGO	Yes	11.54	Unaffordable	Yes	NIA
Libya	3.59	Private	Yes	40.37	Unaffordable	Public	Unavailable	Unavailable	Unavailable	Unavailable	Unavailable
Malawi	1.92	Private	Unavailable	Unavailable	Unavailable	Public	Unavailable	Unavailable	Unavailable	Unavailable	Unavailable
Malaysia	12.88	Private	Yes	12.40	Yes	Public	Unavailable	Unavailable	Unavailable	Unavailable	Unavailable
Mali	2.46	Private	Unavailable	Unavailable	Unavailable	Public	Unavailable	Unavailable	Unavailable	Unavailable	Unavailable
Mexico	15.07	Private	Yes	74.25	Unaffordable	Private	Unavailable	Unavailable	Unavailable	NIA	NIA
Moçambique	4.80	Private	Unavailable	Unavailable	Unavailable	Public	Unavailable	Unavailable	Unavailable	Unavailable	Unavailable
Mongolia	4.85	Private	Unavailable	Unavailable	Unavailable	Public	Yes	22.20	Unaffordable	Unavailable	Unavailable
Nepal	4.56	Private	Yes	8.54	Unaffordable	Public	Yes	4.08	Yes	Unavailable	Unavailable
Niger	2.31	Private	Yes	79.72	Unaffordable	Public	Unavailable	Unavailable	Unavailable	Unavailable	Unavailable
Nigeria	2.77	Private	Yes	10.92	Unaffordable	Public	Unavailable	Unavailable	Unavailable	Unavailable	Unavailable
North Macedonia	17.37	Public	Yes	50.48	Unaffordable	Public	Unavailable	Unavailable	Unavailable	Unavailable	Unavailable
Pakistan	4.23	Public	Yes	0.00	Yes	Public	Yes	6.60	Unaffordable	Unavailable	Unavailable
Peru	9.90	Private	Yes	47.72	Unaffordable	Public	Unavailable	Unavailable	Unavailable	Unavailable	Unavailable
Romania	20.32	Private	Yes	27.30	Unaffordable	Public	Yes	29.94	Unaffordable	Unavailable	Unavailable
Rwanda	0.09	Private	Yes	22.30	Unaffordable	Public	Unavailable	Unavailable	Unavailable	NIA	NIA
Senegal	3.34	Private	Yes	25.87	Unaffordable	Public	Unavailable	Unavailable	Unavailable	Unavailable	Unavailable
Serbia	16.35	Private	Unavailable	Unavailable	Unavailable	Public	Yes	26.20	Unaffordable	Unavailable	Unavailable
Somalia	0.10	Private	Unavailable	Unavailable	Unavailable	Private	Yes	15.43	Unaffordable	NIA	NIA
South Africa	9.37	Private	Unavailable	Unavailable	Unavailable	Public	Yes	7.14	Yes	Yes	7.14
South Sudan	5.05	Private	Yes	14.00	Unaffordable	Unavailable	NIA	NIA	NIA	NIA	NIA
Sri Lanka	1.35	Private	Yes	5.70	Unaffordable	Public	Unavailable	Unavailable	Unavailable	Unavailable	Unavailable
Sudan	0.03	Private	Yes	19.20	Unaffordable	Public	Unavailable	Unavailable	Unavailable	Unavailable	Unavailable
Syria	0.55	Private	Yes	1.24	Unaffordable	Public	Yes	0.00	Yes	Yes	1.05
Tanzania	2.18	Private	Yes	13.20	Unaffordable	Public	Yes	12.40	Unaffordable	Unavailable	Unavailable

Stolbrink M, *et al. Thorax* 2024; 79:676–679. doi: 10.1136/thorax-2023-221349

Appendix – Availability, cost, and affordability of essential medicines for chronic respiratory diseases in low- and middle-income countries: a cross-sectional study – Stolbrink et al.

Thailand	9.05	Public	Unavailable	Unavailable	Unavailable	Public	Unavailable	Unavailable	Unavailable	NIA	NIA
The Gambia	0.91	Private	Unavailable	Unavailable	Unavailable	Public	Unavailable	Unavailable	Unavailable	Unavailable	Unavailable
Timor Leste	4.43	Unavailable	NIA	NIA	NIA	Private	Unavailable	Unavailable	Unavailable	NIA	NIA
Tunisia	4.74	Public	Yes	24.20	Unaffordable	Public	Unavailable	Unavailable	Unavailable	Yes	NIA
Turkiye	13.90	Private	Unavailable	Unavailable	Unavailable	Private	Unavailable	Unavailable	Unavailable	Unavailable	Unavailable
Uganda	0.07	Private	Unavailable	Unavailable	Unavailable	NGO	Yes	NIA	NIA	Unavailable	Unavailable
Venezuela	0.94	Private	Yes	27.57	Unaffordable	Private	Unavailable	Unavailable	Unavailable	NIA	NIA
Viet Nam	6.00	Private	Yes	20.80	Unaffordable	Public	Yes	22.40	Unaffordable	Unavailable	Unavailable
Yemen	3.23	Public	Unavailable	Unavailable	Unavailable	Private	Unavailable	Unavailable	Unavailable	NIA	NIA
Zambia	2.59	Private	Unavailable	Unavailable	Unavailable	Public	Unavailable	Unavailable	Unavailable	Unavailable	Unavailable
Zimbabwe	11.58	Private	Unavailable	Unavailable	Unavailable	Public	Unavailable	Unavailable	Unavailable	Unavailable	Unavailable

Stolbrink M, *et al. Thorax* 2024; 79:676–679. doi: 10.1136/thorax-2023-221349

Appendix – Availability, cost, and affordability of essential medicines for chronic respiratory diseases in low- and middle-income countries: a cross-sectional study – Stolbrink et al.

Supplementary Table 7: Availability, cost, and affordability of ICS-LABA (100+6mcg/dose) by country and type of facility.

Cost is for standardised dose and formulation (inhaled budesonide-formoterol or beclomethasone-formoterol combination 100+6 mcg/dose, 120 doses). CMS costs are wholesale costs, unsuitable for affordability calculations. Affordable: one month's treatment costs less than one day's wage of national minimum wage. $: US$; ICS-LABA: inhaled corticosteroid-long-acting beta-agonist combination; HCF: Healthcare facility; CMS: Central medicine stores; NIA: No information available; DRC: Democratic Republic of Congo; NGO: Non-governmental organisation.

(1): salmeterol-fluticasone 50+1500mcg/dose available in pharmacy; (2): salmeterol-fluticasone 25+250 mcg/dose available in HCF; (3): salmeterol-fluticasone 50+500 mcg/dose available in pharmacy; (4): salmeterol-fluticasone 50+500 available in CMS; (5): salmeterol-fluticasone 50+100, 50+250 and 50+500 mcg/dose available in HCF; (6): salmeterol-fluticasone 50+250 mcg/dose available in pharmacy; (7): salmeterol-fluticasone 50+250 mcg/dose available in pharmacy; (8): salmeterol-fluticasone 36+250 mcg/dose available in pharmacy; (9): fluticasone-formoterol 5+125 mcg/dose available in HCF; (10): salmeterol-fluticasone 25+125 mcg/dose available in HCF and CMS; (11): salmeterol-fluticasone 25+50 mcg/dose available in CMS; (12): salmeterol-fluticasone 25+125 mcg/dose available in CMS; (13): salmeterol-fluticasone 50+100 mcg/dose available in pharmacy; (14): salmeterol-fluticasone 25+250 mcg/dose available in HCF and CMS; (15): salmeterol-fluticasone 50+250 mcg/dose available in pharmacy; (16): salmeterol-fluticasone 25+125 mcg/dose available in HCF; (17): salmeterol-fluticasone 25+50, 25+125, 25+250 mcg/dose available in HCF; (18): salmeterol-fluticasone 25+125 mcg/dose available in pharmacy; (19): salmeterol-fluticasone 50+250 mcg/dose available in pharmacy.

Country	Minimum daily wage ($)	Pharmacy				HCF				CMS	
		Type of facility	ICS-LABA (100+6) available?	ICS-LABA (100+6) cost ($)	ICS-LABA (100+6) affordable?	Type of facility	ICS-LABA (100+6) available?	ICS-LABA (100+6) cost ($)	ICS-LABA (100+6) affordable?	ICS-LABA (100+6) available?	ICS-LABA (100+6) cost ($)
Albania	11.09	Private	Yes	0.00	Yes	Public	Yes	34.34	Unaffordable	NIA	NIA
Algeria	5.39	Private	Unavailable (1)	Unavailable	Unavailable	Public	Unavailable	Unavailable	Unavailable	Unavailable	Unavailable
Angola	2.46	Private	Unavailable	Unavailable	Unavailable	Public	Unavailable	Unavailable	Unavailable	Unavailable	Unavailable
Argentina	15.60	Private	Unavailable	Unavailable	Unavailable	Public	Unavailable (2)	Unavailable	Unavailable	Unavailable	Unavailable
Bosnia and Herzegovina	10.89	Private	Yes	44.29	Unaffordable	Public	Unavailable	Unavailable	Unavailable	NIA	NIA
Brazil	8.86	Private	Yes	22.64	Unaffordable	Public	Unavailable	Unavailable	Unavailable	Unavailable	Unavailable
Burkina Faso	2.13	Private	Unavailable	Unavailable	Unavailable	Public	Unavailable	Unavailable	Unavailable	NIA	NIA
Cameroon	2.23	Private	Yes	19.20	Unaffordable	Public	Yes	18.32	Unaffordable	Unavailable	Unavailable
Chad	3.70	NIA	NIA	NIA	NIA	NIA	NIA	NIA	NIA	Unavailable	Unavailable
China	10.62	Private	Unavailable (3)	Unavailable	Unaffordable	Public	Unavailable	Unavailable	Unavailable	Unavailable (4)	Unavailable
DRC	3.54	Private	Yes	10.00	Unaffordable	Public	Yes	10.50	Unaffordable	Unavailable	Unavailable
Ecuador	15.17	Private	Yes	19.00	Unaffordable	Private	Unavailable	Unavailable	Unavailable	Unavailable	Unavailable
Egypt	5.85	Private	Yes	5.60	Yes	Private	Unavailable (5)	Unavailable	Unavailable	Unavailable	Unavailable

Stolbrink M, *et al. Thorax* 2024; 79:676–679. doi: 10.1136/thorax-2023-221349

Appendix – Availability, cost, and affordability of essential medicines for chronic respiratory diseases in low- and middle-income countries: a cross-sectional study – Stolbrink et al.

Eswatini	0.92	Private	Unavailable (6)	Unavailable	Unavailable	NIA	NIA	NIA	NIA	Unavailable	Unavailable
Ethiopia	0.31	Private	Unavailable	Unavailable	Unavailable	Public	Unavailable	Unavailable	Unavailable	Unavailable	Unavailable
Ghana	1.19	Private	Unavailable (7)	Unavailable	Unavailable	Public	Unavailable	Unavailable	Unavailable	Unavailable	Unavailable
Guinea	2.44	Private	Unavailable	Unavailable	Unavailable	NIA	NIA	NIA	NIA	Unavailable	Unavailable
Honduras	14.82	Private	Unavailable	Unavailable	Unavailable	Public	Unavailable	Unavailable	Unavailable	NIA	NIA
India	2.14	Private	Yes	3.39	Unaffordable	Public	Unavailable	Unavailable	Unavailable	Unavailable	Unavailable
Indonesia	7.22	Private	Yes	19.71	Unaffordable	Public	Yes	28.00	Unaffordable	Yes	13.31
Iran	20.44	Private	Unavailable	Unavailable	Unavailable	Public	Unavailable	Unavailable	Unavailable	Unavailable	Unavailable
Iraq	9.43	Private	Unavailable	Unavailable	Unavailable	Public	Unavailable	Unavailable	Unavailable	NIA	NIA
Kenya	5.25	Private	Unavailable	Unavailable	Unavailable	Public	Unavailable	Unavailable	Unavailable	NIA	NIA
Kyrgyzstan	0.88	Private	Unavailable (8)	Unavailable	Unavailable	Public	Unavailable	Unavailable	Unavailable	Unavailable	Unavailable
Lesotho	3.60	NIA	NIA	NIA	NIA	NGO	Yes	11.54	Unaffordable	Unavailable	Unavailable
Libya	3.59	Private	Unavailable	Unavailable	Unavailable	Public	Unavailable	Unavailable	Unavailable	Unavailable	Unavailable
Malawi	1.92	Private	Unavailable	Unavailable	Unavailable	Public	Unavailable	Unavailable	Unavailable	Unavailable	Unavailable
Malaysia	12.88	Private	Unavailable	Unavailable	Unavailable	Public	Unavailable (9)	Unavailable	Unavailable	Yes	26.48
Mali	2.46	Private	Unavailable	Unavailable	Unavailable	Public	Unavailable	Unavailable	Unavailable	Unavailable	Unavailable
Mexico	15.07	Private	Yes	73.65	Unaffordable	Private	Yes	29.00	Unaffordable	NIA	NIA
Moçambique	4.80	Private	Yes	77.06	Unaffordable	Public	Unavailable	Unavailable	Unavailable	Unavailable	Unavailable
Mongolia	4.85	Private	Yes	33.60	Unaffordable	Public	Yes	22.20	Unaffordable	Yes	29.85
Nepal	4.56	Private	Unavailable	Unavailable	Unavailable	Public	Unavailable	Unavailable	Unavailable	Unavailable	Unavailable
Niger	2.31	Private	Yes	81.46	Unaffordable	Public	Unavailable	Unavailable	Unavailable	Unavailable	Unavailable
Nigeria	2.77	Private	Unavailable	Unavailable	Unavailable	Public	Unavailable (10)	Unavailable	Unavailable	Unavailable	Unavailable (10)
North Macedonia	17.37	Public	Unavailable	Unavailable	Unavailable	Public	Unavailable	Unavailable	Unavailable	Yes	57.67
Pakistan	4.23	Public	Yes	0.00	Yes	Public	Yes	4.84	Unaffordable	Unavailable (11)	Unavailable
Peru	9.90	Private	Yes	42.12	Unaffordable	Public	Unavailable	Unavailable	Unavailable	Unavailable	Unavailable
Romania	20.32	Private	Unavailable	Unavailable	Unavailable	Public	Yes	14.66	Yes	Unavailable (12)	Unavailable
Rwanda	0.09	Private	Unavailable	Unavailable	Unavailable	Public	Unavailable	Unavailable	Unavailable	NIA	NIA
Senegal	3.34	Private	Yes	19.29	Unaffordable	Public	Unavailable	Unavailable	Unavailable	Unavailable	Unavailable
Serbia	16.35	Private	Yes	NIA	NIA	Public	Unavailable	Unavailable	Unavailable	Yes	NIA

Stolbrink M, *et al. Thorax* 2024; 79:676–679. doi: 10.1136/thorax-2023-221349

Appendix – Availability, cost, and affordability of essential medicines for chronic respiratory diseases in low- and middle-income countries: a cross-sectional study – Stolbrink et al.

Somalia	0.10	Private	Unavailable	Unavailable	Unavailable	Private	Unavailable	Unavailable	Unavailable	NIA	NIA
South Africa	9.37	Private	Unavailable (13)	Unavailable	Unavailable	Public	Unavailable	Unavailable	Unavailable	Unavailable	Unavailable
South Sudan	5.05	Private	Yes	42.00	Unaffordable	Public	NIA	NIA	NIA	NIA	NIA
Sri Lanka	1.35	Private	Unavailable	Unavailable	Unavailable	Public	Unavailable (14)	Unavailable	Unavailable	Unavailable (14)	Unavailable
Sudan	0.03	Private	Unavailable	Unavailable	Unavailable	Public	Unavailable	Unavailable	Unavailable	Unavailable	Unavailable
Syria	0.55	Private	Unavailable	Unavailable	Unavailable	Public	Unavailable	Unavailable	Unavailable	Yes	0.92
Tanzania	2.18	Private	Yes	12.00	Unaffordable	Public	Unavailable	Unavailable	Unavailable	Unavailable	Unavailable
Thailand	9.05	Public	Unavailable (15)	Unavailable	Unavailable	Public	Unavailable (16)	Unavailable	Unavailable	NIA	NIA
The Gambia	0.91	Private	Yes	21.84	Unaffordable	Public	Unavailable	Unavailable	Unavailable	Unavailable	Unavailable
Timor Leste	4.43	NIA	NIA	NIA	NIA	Private	Unavailable	Unavailable	Unavailable	NIA	NIA
Tunisia	4.74	Public	Yes	18.84	Unaffordable	Public	Unavailable (17)	Unavailable	Unavailable	Yes	NIA
Turkiye	13.90	Private	Unavailable (18)	Unavailable	Unavailable	Private	Unavailable	Unavailable	Unavailable	Unavailable	Unavailable
Uganda	0.07	Private	Unavailable	Unavailable	Unavailable	NGO	Yes	13.50	Unaffordable	Unavailable	Unavailable
Venezuela	0.94	Private	Yes	25.92	Unaffordable	Private	Unavailable	Unavailable	Unavailable	NIA	NIA
Viet Nam	6.00	Private	Unavailable	Unavailable	Unavailable	Public	Unavailable	Unavailable	Unavailable	Unavailable	Unavailable
Yemen	3.23	Public	Unavailable	Unavailable	Unavailable	Private	Unavailable	Unavailable	Unavailable	NIA	NIA
Zambia	2.59	Private	Unavailable (19)	Unavailable	Unavailable	Public	Unavailable	Unavailable	Unavailable	Unavailable	Unavailable
Zimbabwe	11.58	Private	Unavailable	Unavailable	Unavailable	Public	Unavailable	Unavailable	Unavailable	Unavailable	Unavailable

Appendix – Availability, cost, and affordability of essential medicines for chronic respiratory diseases in low- and middle-income countries: a cross-sectional study – Stolbrink et al.

Supplementary Table 8: Availability, cost, and affordability of LAMA by country and type of facility.

Cost is for standardised dose and formulation. Affordable: one month's treatment costs less than one day's wage of national minimum wage. CMS costs are wholesale costs, unsuitable for affordability calculations. Affordable: one month's treatment costs less than one day's wage of national minimum wage. $: US$; LAMA: Long-acting muscarinic antagonist inhaler; HCF: Healthcare facility; CMS: Central medicine stores; No information: NIA available; DRC: Democratic Republic of Congo; NGO: Non-governmental organisation.

(1): Seebri (glycopyrronium) 50 mcg/dose available in pharmacy and HCF; (2): Incruse (umeclidinium) 62.5 mcg/dose available in CMS; (3): Seebri (glycopyrronium) 50 mcg/dose available in HCF and CMS.

Country	Minimum daily wage (US$)	Pharmacy				HCF				CMS	
		Type of facility	LAMA available?	LAMA cost ($)	LAMA affordable?	Type of facility	LAMA available?	LAMA cost ($)	LAMA affordable?	LAMA available?	LAMA cost ($)
Albania	11.09	Private	Unavailable (1)	Unavailable	Unavailable	Public	Unavailable (1)	Unavailable	Unavailable	NIA	NIA
Algeria	5.39	Private	Yes	9.45	Unaffordable	Public	Unavailable	Unavailable	Unavailable	Unavailable	Unavailable
Angola	2.46	Private	Unavailable	Unavailable	Unavailable	Public	Unavailable	Unavailable	Unavailable	Yes	69.04
Argentina	15.60	Private	Yes	104.97	Unaffordable	Public	Yes	63.21	Unaffordable	Unavailable	Unavailable
Bosnia and Herzegovina	10.89	Private	Unavailable	Unavailable	Unavailable	Public	Unavailable	Unavailable	Unavailable	NIA	NIA
Brazil	8.86	Private	Yes	50.97	Unaffordable	Public	Unavailable	Unavailable	Unavailable	Unavailable	Unavailable
Burkina Faso	2.13	Private	Unavailable	Unavailable	Unavailable	Public	Unavailable	Unavailable	Unavailable	NIA	NIA
Cameroon	2.23	Private	Unavailable	Unavailable	Unavailable	Public	Unavailable	Unavailable	Unavailable	Unavailable	Unavailable
Chad	3.70	NIA	NIA	NIA	NIA	NIA	NIA	NIA	NIA	Unavailable	Unavailable
China	10.62	Private	Yes	36.64	Unaffordable	Public	Yes	25.98	Unaffordable	Yes	25.74
DRC	3.54	Private	Unavailable	Unavailable	Unavailable	Public	Unavailable	Unavailable	Unavailable	Unavailable	Unavailable
Ecuador	15.17	Private	Yes	42.37	Unaffordable	Private	Unavailable	Unavailable	Unavailable	Unavailable	Unavailable
Egypt	5.85	Private	Yes	10.24	Unaffordable	Private	Yes	20.28	Unaffordable	Yes	10.23
Eswatini	0.92	Private	Unavailable	Unavailable	Unavailable	NIA	NIA	NIA	NIA	Unavailable	Unavailable
Ethiopia	0.31	Private	Unavailable	Unavailable	Unavailable	Public	Unavailable	Unavailable	Unavailable	Unavailable	Unavailable
Ghana	1.19	Private	Unavailable	Unavailable	Unavailable	Public	Unavailable	Unavailable	Unavailable	Unavailable	Unavailable
Guinea	2.44	Private	Unavailable	Unavailable	Unavailable	NIA	NIA	NIA	NIA	Unavailable	Unavailable
Honduras	14.82	Private	Unavailable	Unavailable	Unavailable	Public	Yes	82.40	Unaffordable	NIA	NIA

Stolbrink M, *et al. Thorax* 2024; 79:676–679. doi: 10.1136/thorax-2023-221349

Appendix – Availability, cost, and affordability of essential medicines for chronic respiratory diseases in low- and middle-income countries: a cross-sectional study – Stolbrink et al.

India	2.14	Private	Yes	2.15	Unaffordable	Public	Unavailable	Unavailable	Unavailable	Unavailable	Unavailable
Indonesia	7.22	Private	Unavailable	Unavailable	Unavailable	Public	Yes	56.00	Unaffordable	yes	36.83
Iran	20.44	Private	Yes	1.00	Yes	Public	Unavailable	Unavailable	Unavailable	Yes	1.00
Iraq	9.43	Private	Unavailable	Unavailable	Unavailable	Public	Unavailable	Unavailable	Unavailable	NIA	NIA
Kenya	5.25	Private	Unavailable	Unavailable	Unavailable	Public	Unavailable	Unavailable	Unavailable	NIA	NIA
Kyrgyzstan	0.88	Private	Unavailable	Unavailable	Unavailable	Public	Yes	26.04	Unaffordable	Unavailable	Unavailable
Lesotho	3.60	NIA	NIA	NIA	NIA	NGO	Unavailable	Unavailable	Unavailable	Unavailable	Unavailable
Libya	3.59	Private	Yes	39.33	Unaffordable	Public	Unavailable	Unavailable	Unavailable	Unavailable	Unavailable
Malawi	1.92	Private	Unavailable	Unavailable	Unavailable	Public	Unavailable	Unavailable	Unavailable	Unavailable	Unavailable
Malaysia	12.88	Private	Yes	47.29	Unaffordable	Public	Yes	13.83	Unaffordable	Unavailable	Unavailable
Mali	2.46	Private	Unavailable	Unavailable	Unavailable	Public	Unavailable	Unavailable	Unavailable	Unavailable	Unavailable
Mexico	15.07	Private	Yes	61.15	Unaffordable	Private	Yes	46.00	Unaffordable	NIA	NIA
Moçambique	4.80	Private	Unavailable	Unavailable	Unavailable	Public	Unavailable	Unavailable	Unavailable	Unavailable	Unavailable
Mongolia	4.85	Private & Public	Unavailable	Unavailable	Unavailable	Public	Unavailable	Unavailable	Unavailable	Unavailable	Unavailable
Nepal	4.56	Private	Yes	3.92	Yes	Public	Yes	3.56	Yes	Unavailable	Unavailable
Niger	2.31	Private	Unavailable	Unavailable	Unavailable	Public	Unavailable	Unavailable	Unavailable	Unavailable	Unavailable
Nigeria	2.77	Private	Yes	133.20	Unaffordable	Public	Unavailable	Unavailable	Unavailable	Unavailable	Unavailable
North Macedonia	17.37	Public	Yes	30.53	Unaffordable	Public	Unavailable	Unavailable	Unavailable	Yes	30.62
Pakistan	4.23	Public	Yes	0.00	Yes	Public	Yes	5.28	Unaffordable	Unavailable (2)	Unavailable
Peru	9.90	Private	Yes	137.20	Unaffordable	Public	Unavailable	Unavailable	Unavailable	Unavailable	Unavailable
Romania	20.32	Private	Yes	27.88	Unaffordable	Public	Yes	29.81	Unaffordable	Yes	NIA
Rwanda	0.09	Private	Unavailable	Unavailable	Unavailable	Public	Unavailable	Unavailable	Unavailable	NIA	NIA
Senegal	3.34	Private	Unavailable	Unavailable	Unavailable	Public	Unavailable	Unavailable	Unavailable	Unavailable	Unavailable
Serbia	16.35	Private	Yes	NIA	NIA	Public	Unavailable	Unavailable	Unavailable	Yes	NIA
Somalia	0.10	Private	Unavailable	Unavailable	Unavailable	Private	Unavailable	Unavailable	Unavailable	NIA	NIA
South Africa	9.37	Private	Yes	39.52	Unaffordable	Public	Unavailable (3)	Unavailable	Unavailable	Unavailable (3)	Unavailable
South Sudan	5.05	Private	Unavailable	Unavailable	Unavailable	NIA	NIA	NIA	NIA	NIA	NIA
Sri Lanka	1.35	Private	Unavailable	Unavailable	Unavailable	Public	Unavailable	Unavailable	Unavailable	Yes	0.92

Appendix – Availability, cost, and affordability of essential medicines for chronic respiratory diseases in low- and middle-income countries: a cross-sectional study – Stolbrink et al.

Sudan	0.03	Private	Unavailable	Unavailable	Unavailable	Public	Unavailable	Unavailable	Unavailable	Unavailable	Unavailable
Syria	0.55	Private	Yes	0.78	Unaffordable	Public	Yes	0	Yes	Yes	0.66
Tanzania	2.18	Private	Yes	14.00	Unaffordable	Public	Unavailable	Unavailable	Unavailable	Unavailable	Unavailable
Thailand	9.05	Public	Unavailable	Unavailable	Unavailable	Public	Yes	15.82	Unaffordable	NIA	NIA
The Gambia	0.91	Private	Yes	83.72	Unaffordable	Public	Unavailable	Unavailable	Unavailable	Unavailable	Unavailable
Timor Leste	4.43	NIA	NIA	NIA	NIA	Private	Unavailable	Unavailable	Unavailable	NIA	NIA
Tunisia	4.74	Public	Yes	22.12	Unaffordable	Public	Yes	22.12	Unaffordable	Yes	NIA
Turkiye	13.90	Private	Yes	14.24	Unaffordable	Private	Unavailable	Unavailable	Unavailable	Unavailable	Unavailable
Uganda	0.07	Private	Unavailable	Unavailable	Unavailable	NGO	Unavailable	Unavailable	Unavailable	Unavailable	Unavailable
Venezuela	0.94	Private	Unavailable	Unavailable	Unavailable	Private	Unavailable	Unavailable	Unavailable	NIA	NIA
Viet Nam	6.00	Private	Yes	32.00	Unaffordable	Public	Yes	33.60	Unaffordable	Unavailable	Unavailable
Yemen	3.23	Public	Yes	0.05	Yes	Private	Yes	28.77	Unaffordable	NIA	NIA
Zambia	2.59	Private	Unavailable	Unavailable	Unavailable	Public	Unavailable	Unavailable	Unavailable	Unavailable	Unavailable
Zimbabwe	11.58	Private	Unavailable	Unavailable	Unavailable	Public	Unavailable	Unavailable	Unavailable	Unavailable	Unavailable

Stolbrink M, *et al. Thorax* 2024; 79:676–679. doi: 10.1136/thorax-2023-221349

Appendix – Availability, cost, and affordability of essential medicines for chronic respiratory diseases in low- and middle-income countries: a cross-sectional study – Stolbrink et al.

Supplementary Table 9: Availability and cost for one month's treatment for other essential medicines

Cost is for standardised dose and formulation. Insufficient data for calculations for prednisolone solution, SABA nebuliser, SABA injection. SAMA: Short-acting muscarinic inhaler; SABA: short-acting beta-agonist; IQR: inter-quartile range. *Oral prednisolone price is for a course of 40mg once a day for 5 days.

		**Medicine**				
**Facility**		**SAMA**	**5mg prednisolone tablets***	**5mg/ml prednisolone solution**	**5mg/ml SABA nebuliser**	**SABA injection**
**Pharmacy**	**Availability**	19/57 (33 %)	47/57 (82%)	3/57 (5%)	15/57 (26%)	5/57 (9%)
	**Median cost (US$)**	11.16	1.65			
	**IQR (US$)**	5.44-10.08	0.60-3.29			
	**Range (US$)**	0-65.90	0.00-8.91			
**HCF**	**Availability**	19/56 (34%)	40/56 (71%)	2/56 (4%)	19/56 (34%)	5/56 (9%)
	**Median cost (US$)**	9.46	0.02			
	**IQR (US$)**	2.26-7.30	0.01-0.08			
	**Range (US$)**	0-71.40	0-1.00			
**CMS**	**Availability**	12/46 (26%)	35/46 (76%)	2/46 (4%)	4/46 (9%)	9/46 (20%)
	**Median cost (US$)**	2.31	0.02			
	**IQR (US$)**	1.67-2.65	0.01-0.03			
	**Range (US$)**	0-8.34	0-0.58			

Stolbrink M, *et al. Thorax* 2024; 79:676–679. doi: 10.1136/thorax-2023-221349

Appendix – Availability, cost, and affordability of essential medicines for chronic respiratory diseases in low- and middle-income countries: a cross-sectional study – Stolbrink et al.

Supplementary Figure 1: World map of LMICs where medicine data was available

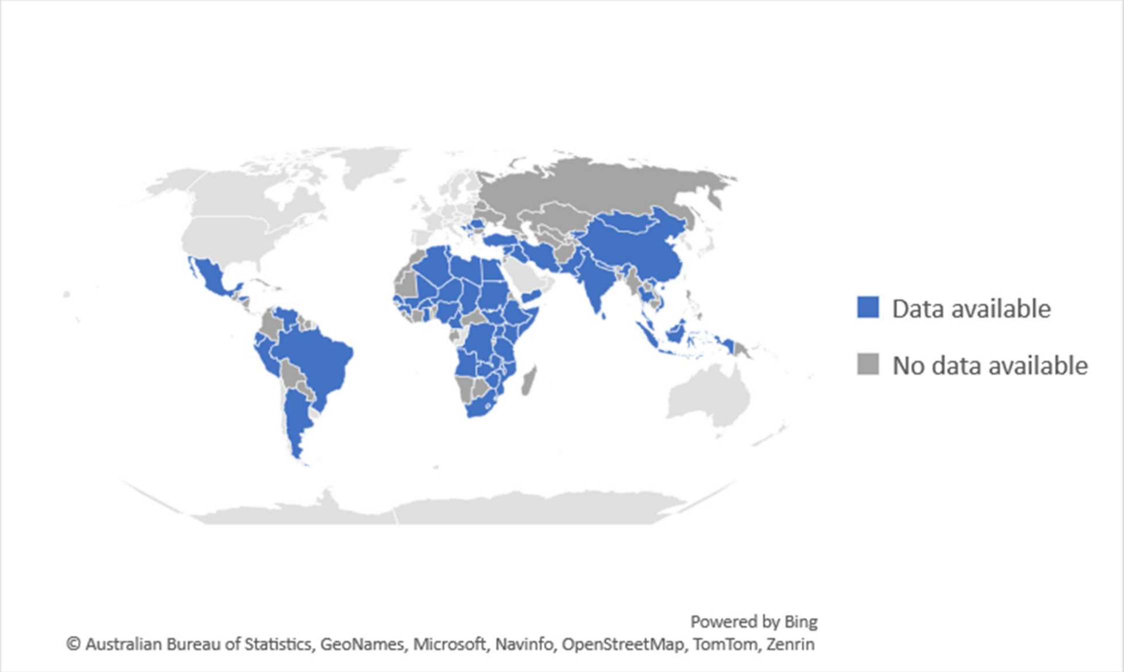

Stolbrink M, *et al. Thorax* 2024; 79:676–679. doi: 10.1136/thorax-2023-221349

Appendix – Availability, cost, and affordability of essential medicines for chronic respiratory diseases in low- and middle-income countries: a cross-sectional study – Stolbrink et al.

Supplementary Figure 2: Box and whisker plots for ranges, interquartile ranges and medians of median 1) costs for one month's treatment in US$ in pharmacies and HCFs (a, b) and 2) days of work (DOW) for one month's treatment in pharmacies and HCFs (c, d).

"+": upper range outside of axis range; maximum range panel a) ICS-LABA (100+6) $81.46, ICS-LABA (200+6) $79.72, LAMA $37.20; b) ICS-LABA (200+6) $79.40, LAMA $82.40; c) SABA 73.4 DOW, ICS 152.8 DOW, ICS-LABA (100+6) 35.2 DOW, ICS-LABA (200+6) 586.8 DOW, LAMA 91.9 DOW, 5 days OCS 48.9 DOW; d) SABA 43.3 DOW, ICS 86.7 DOW, ICS-LABA (100+6) 194.9 DOW, ICS-LABA (200+6) 154.3 DOW, LAMA 29.6 DOW. Cost for OCS is 5 days course of oral prednisolone, 40mg once a day using 5mg tablets. SABA: Short-acting beta-agonist inhaler; ICS: Inhaled corticosteroid; ICS-LABA: inhaled corticosteroid-long-acting beta-agonist (formoterol) combination; LAMA: Long-acting muscarinic antagonist inhaler; HCF: Healthcare facility; CMS: Central medicine stores; DOW: days of work required to pay for one month's treatment; OCS: oral corticosteroids.

Stolbrink M, *et al. Thorax* 2024; 79:676–679. doi: 10.1136/thorax-2023-221349

Appendix – Availability, cost, and affordability of essential medicines for chronic respiratory diseases in low- and middle-income countries: a cross-sectional study – Stolbrink et al.

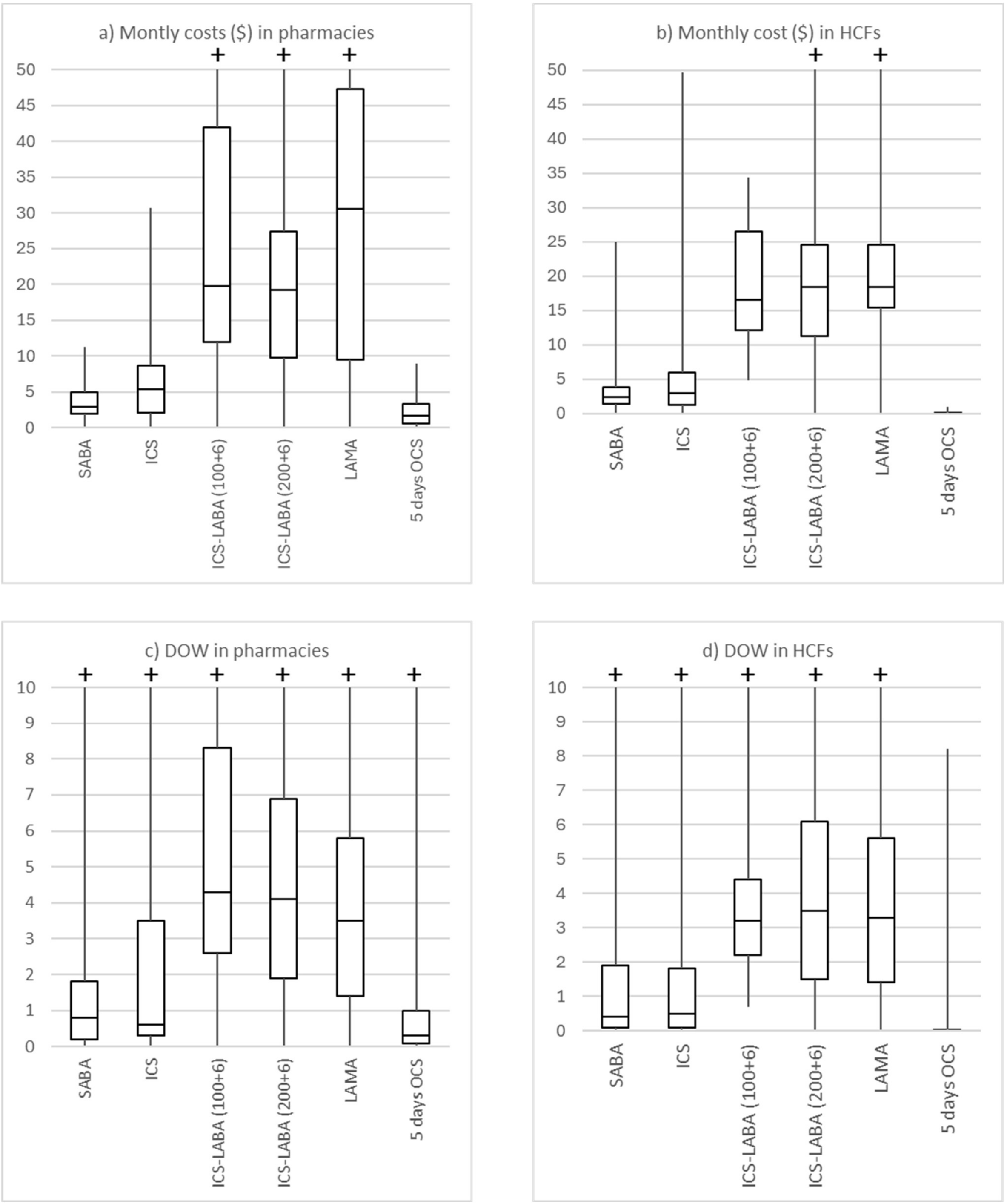

Stolbrink M, *et al. Thorax* 2024; 79:676–679. doi: 10.1136/thorax-2023-221349

Appendix – Availability, cost, and affordability of essential medicines for chronic respiratory diseases in low- and middle-income countries: a cross-sectional study – Stolbrink et al.

Supplementary Figure 3: Median costs for one month's treatment in US$ or days of work for one month's treatment by WHO region and World Bank Income Group, in pharmacies (a-d) and HCF (e-h).

Cost for OCS is 5 days course of oral prednisolone, 40mg once a day using 5mg tablets. SABA: Short-acting beta-agonist inhaler; ICS: Inhaled corticosteroid; ICS-LABA: inhaled corticosteroid-long-acting beta-agonist (formoterol) combination; LAMA: Long-acting muscarinic antagonist inhaler; HCF: Healthcare facility; CMS: Central medicine stores; DOW: days of work required to pay for one month's treatment; OCS: oral corticosteroids.

Stolbrink M, *et al. Thorax* 2024; 79:676–679. doi: 10.1136/thorax-2023-221349

Appendix – Availability, cost, and affordability of essential medicines for chronic respiratory diseases in low- and middle-income countries: a cross-sectional study – Stolbrink et al.

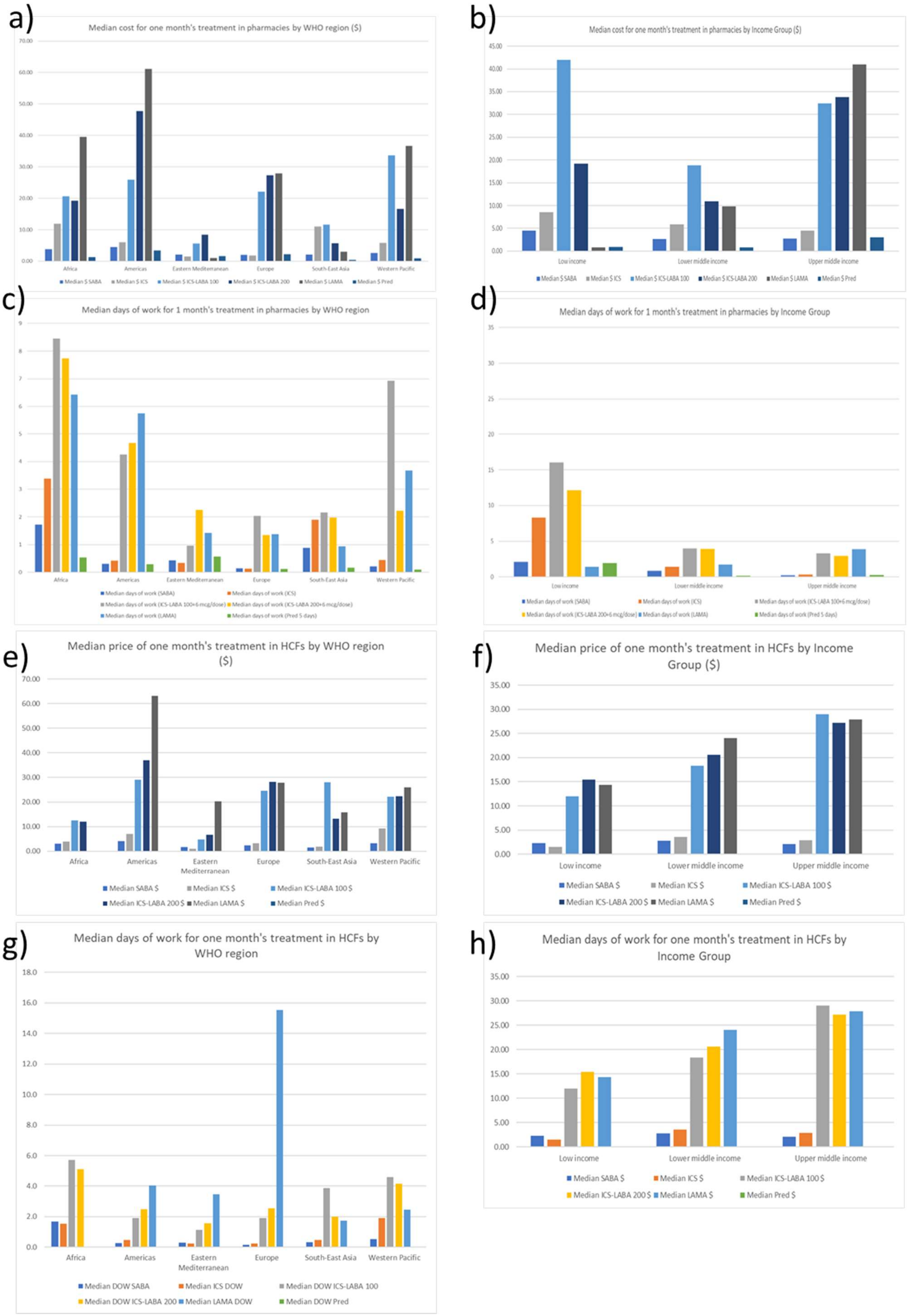

Stolbrink M, *et al. Thorax* 2024; 79:676–679. doi: 10.1136/thorax-2023-221349

Appendix – Availability, cost, and affordability of essential medicines for chronic respiratory diseases in low- and middle-income countries: a cross-sectional study – Stolbrink et al.

Supplementary Figure 4: Comparison of originator and generic medicine costs

Figure shows whether generic or originator is cheaper in a) pharmacy, b) HCF, c) CMS. Number of LMICs where generic and originators of given drug class were available.

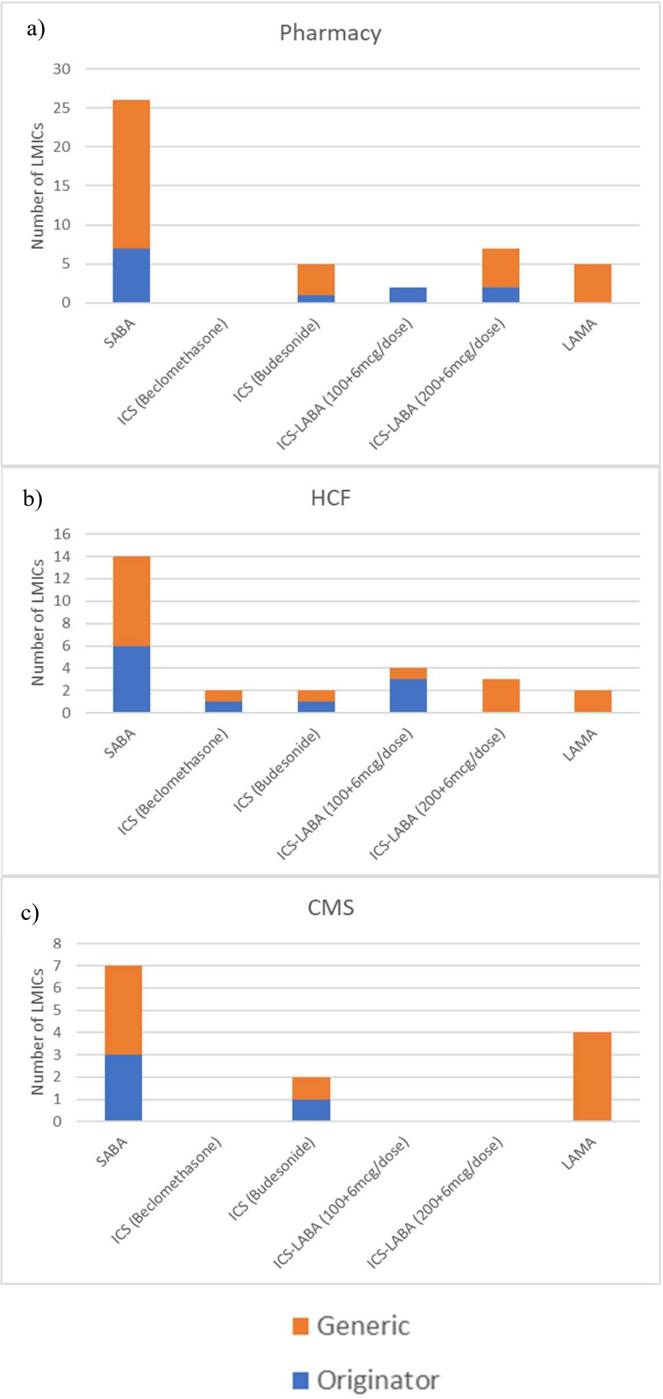

Appendix – Availability, cost, and affordability of essential medicines for chronic respiratory diseases in low- and middle-income countries: a cross-sectional study – Stolbrink et al.

References

1 Elm E von, Altman DG, Egger M, *et al.* The Strengthening the Reporting of Observational Studies in Epidemiology (STROBE) statement: guidelines for reporting observational studies. https://core.ac.uk/reader/33050540?utm_source=linkout (accessed Aug 23, 2023).

2 KoBoToolbox. 2020; published online Nov 2. https://www.kobotoolbox.org/ (accessed Nov 2, 2020).

3 Babar Z-U-D, Lessing C, Mace C, Bissell K. The Availability, Pricing and Affordability of Three Essential Asthma Medicines in 52 Low- and Middle-Income Countries. *PharmacoEconomics* 2013; **31**: 1063–82.

4 World Health Organization, Health Action International. Measuring medicine prices, availability, affordability and price components - Second edition. Geneva, 2008.

5 World Health Organization. World Health Organization Model List of Essential Medicines - 22nd List. Geneva: World Health Organization, 2021.

6 World Bank. World Bank Country and Lending Groups. 2023; published online July 11. https://datahelpdesk.worldbank.org/knowledgebase/articles/906519-world-bank-country-and-lending-groups (accessed July 11, 2023).

7 Exchange rates. Exchange rates. https://www.exchangerates.org.uk (accessed April 28, 2023).

8 International Labour Organization. International Labour Organization - Statistics on Wages. Geneva, Switzerland: United Nations, 2023 https://ilostat.ilo.org/topics/wages/.

9 World Health Organization. Regional offices. 2023. https://www.who.int/about/who-we-are/regional-offices (accessed Sept 1, 2023).
